# Exploiting evolutionary diversity of cellulose synthase catalytic subunits to generate novel cellulose microfibril structure in Arabidopsis

**DOI:** 10.1093/jxb/eraf511

**Published:** 2025-11-21

**Authors:** Manoj Kumar, Leonardo D Gomez, Laura Faas, Simon Turner

**Affiliations:** University of Manchester, Faculty of Biology, Medicine and Health; Michael Smith Building, Manchester M13 9PT, UK; Centre of Novel Agricultural Products (CNAP), Department of Biology, University of York, York YO10 5DD, UK; Centre of Novel Agricultural Products (CNAP), Department of Biology, University of York, York YO10 5DD, UK; University of Manchester, Faculty of Biology, Medicine and Health; Michael Smith Building, Manchester M13 9PT, UK; University of Antwerp, Belgium

**Keywords:** Arabidopsis, bryophytes, cellulose, CESA protein, charophycean green algae, lycophyte, secondary cell wall, seed plants

## Abstract

Cellulose is pivotal in regulating plant cell size and shape, and represents an abundant renewable resource for producing materials and chemicals. In seed plants, cellulose is synthesized at the plasma membrane by a hexameric protein complex synthesizing 18 glucose chains that bond together to form a microfibril; however, significant variation exists in the structure and physical properties of cellulose synthesized by other species and between different cell types. In this study, we surveyed the ability of 15 different catalytic subunits of the cellulose synthase complex (CESA proteins) derived from four species of charophycean green algae, a lycophyte, a bryophyte, and a fern to synthesize cellulose in the Arabidopsis secondary cell walls. Several CESA proteins can function in Arabidopsis in conjunction with endogenous CESA proteins in a pattern not easily predictable based on phylogenetics, demonstrating that heterologous expression is a valuable functional analysis tool. Additionally, two moss CESA proteins synthesized cellulose without Arabidopsis CESAs. The cellulose produced by the moss CESA proteins exhibited a much higher proportion of surface-exposed glucose residues but was sufficient to support normal plant growth. This study demonstrates that heterologous expression of CESA proteins generates cellulose with novel structures that offer a more suitable feedstock for biotechnological applications.

## Teaser

CESA proteins from diverse green plants were examined for their ability to synthesise cellulose in Arabidopsis. Proteins from moss were able to function, albeit synthesizing cellulose with lower crystallinity.

## Introduction

Global warming caused by anthropogenic emissions of greenhouse gasses has generated a dramatic increase in demand for materials and chemicals from renewable resources (de Vries *et al*., 2021). Plant biomass is an abundant renewable feedstock with the potential to satisfy this demand, making it one of the few resources able to make a meaningful contribution towards reducing global CO_2_ emissions. Consequently, there has been considerable investment in generating biofuels and chemicals using plant cell walls as a feedstock (de Vries *et al*., 2021). Recent progress in developing novel forms of engineered wood that include flexible and mouldable wood (Xiao *et al*., 2021), densified wood (Song *et al*., 2018), and even transparent wood (Jia *et al*., 2019) has dramatically improved the mechanical properties of plant biomass, allowing it to be used in a much wider range of applications.

Cellulose is the principal constituent of plant biomass and is composed of unbranched chains of β-1,4-glucose that associate and form cellulose microfibrils that confer remarkable structural properties to this polymer. Improving sugar release from cellulose and other plant cell wall polysaccharides is a significant target for improving plant biomass as feedstock for the renewable production of cellulosic ethanol, other biofuels, and chemicals. In addition, various materials are generated by breaking down cellulose microfibrils, including microfibrillated cellulose and cellulose nanocrystals, which have a wide range of uses in producing novel biomaterials (Ding *et al*., 2023; Schubert *et al*., 2023). The properties of these novel products depend upon the properties and composition of the cellulose–hemicellulose matrix of the plant cell wall. It is likely that additional improvements in the feedstock properties for producing biomaterials and biofuels could be attained if a wider variety of cellulose microfibrils with clearly altered structural characteristics could be generated *in planta*.

In plants, the catalytic subunits of the cellulose synthase complex (CSC), the protein complex responsible for the polymerization of UDP-glucose into cellulose, are commonly known as the CESA proteins. In land plants (embryophytes), the CESA proteins form an 18-mer (Fernandes *et al*., 2011; Purushotham *et al*., 2020; Cosgrove, 2024) and constitute a hexagonal-shaped complex, known as a rosette, that synthesizes a cellulose microfibril as it moves through the plane of the plasma membrane. Apart from embryophytes, cellulose is produced by a diverse group of organisms, including green algae (charophytes and chlorophytes), rhodophytes (red algae), xanthophytes (yellow-green algae), phaeophytes (brown algae), oomycetes, dinoflagellates, slime moulds, tunicates, and bacteria (Niklas, 2004). There is wide variation in the arrangement and number of the catalytic subunits within the complex synthezising cellulose that generates enormous variation in the structure of the cellulose microfibril synthesized (Tsekos, 1999). This variation represents an untapped source of structurally different cellulose with the potential for developing new biomaterials with improved chemical and physical properties. Despite the potential, work in this area is minimal. The current state of the art involves a CESA protein from the marine red alga *Calliarthron tuberculosum* (CtCESA1) that contains an additional carbohydrate-binding domain (CBD48) not found in any CESA proteins from green plants. CtCESA1 exhibited cellulose synthase activity *in vitro* and demonstrated a small but significant complementation of the dark-grown hypocotyl defect of the Arabidopsis primary cell wall cellulose mutant, *cesa6^prc^*; however, there was no demonstration of any complementation of the cellulose-deficient phenotype (Xue *et al*., 2022).

We undertook a comprehensive study by expressing 15 different CESA genes from a diverse range of plant species known to synthesize cellulose using a hexameric rosette structure. We tested their ability to complement the Arabidopsis *cesa* secondary cell wall mutants. We tested if they are functionally equivalent to those of Arabidopsis CESAs and whether they can function independently to synthesize cellulose in the absence of any of the Arabidopsis secondary cell wall CESA proteins. We demonstrate that heterologous expression of CESA proteins is useful for functional analysis of CESA proteins and represents a means of generating variation in the properties of the cellulose produced. It represents the first demonstration of how heterologous expression of different CESA proteins can generate variation in the structure and properties of cellulose microfibrils.

## Materials and methods

### Sequence retrieval

Three main strategies were used for building the collection of GT2 sequences, literature searches, BLAST searches, and keyword searches. For the literature search, sequences described in Blum *et al*. (2012) (oomycete CESA sequences), Brawley *et al*. (2017), Chan *et al*. (2019), Little *et al*. (2018), Pang *et al*. (2020), Sena *et al*. (2019), Wan *et al*. (2018), and Wang *et al*. (2020) were retrieved. For BLAST searches, 40 Arabidopsis GT2 sequences and a selection of 15 bacterial BcsA sequences were used as queries to search fully sequenced genomes at Phytozome (Goodstein *et al*., 2012). A local database was created to search fully sequenced genomes publicly available but not included in Phytozome. This database included genomes from gymnosperms (*Cycas micholitzii*, *Ginkgo biloba*, *Gnetum montanum*, *Picea abies*, *Picea glauca*, *Pseudotsuga menziesii*, *Pinus pinaster*, *Picea sitchensis*, *Pinus sylvestris*, and *Pinus taeda* all downloaded from https://bioinformatics.psb.ugent.be/plaza/versions/gymno-plaza/), ferns [*Azolla filiculoides* (Li *et al*., 2018), *Salvinia cucullate* (Li *et al*., 2018), *Ceratopteris richardii* (Marchant *et al*., 2019), and *Adiantum capillus-veneris* (Fang *et al*., 2022)], bryophytes [*Anthoceros angustus* (Zhang *et al*., 2020), *Anthoceros agrestis* Bonn, *Anthoceros agrestis* Oxford, and *Anthoceros punctatus* (Li *et al*., 2020)], and charophycean green algae [CGA; *Mesotaenium endlicherianum* (Wang *et al*., 2020), *Spirogloea muscicola* (Cheng *et al*., 2019), *Chara braunii* (Nishiyama *et al*., 2018), *Klebsormidium nitens* (Hori *et al*., 2014), *Chlorokybus atmophyticus* (Wang *et al*., 2020), and *Mesostigma viride* (Liang *et al*., 2020)]. Protein sequences were downloaded and BLAST searched with the same set of query sequences as above using a local copy of the BLASTP program (Camacho *et al*., 2009). Further BLAST searches were made in the NCBI reference sequences (RefSeq) database (Pruitt *et al*., 2007) and the 1 kp plant transcriptome database (One Thousand Plant Transcriptome Initiative, 2019). These searches were performed at the CNGB server, https://db.cngb.org/blast/blast/blastp/. For keyword searches, PFMAM domain IDs, PF03552 (Cellulose_synt), PF00535 (Glycos_transf_2), PF13632 (Glyco_*trans*_2_3), and PF14569 (zf-UDP) were searched at the Phytozome and Uniport database servers.

### Sequence analysis

All sequences were retrieved and stored in Microsoft Excel. Simple analyses such as identifying the starting amino acid, determining the protein length, and searching for the presence of catalytic motifs, DD, DxD, ED, QxxRW, and FxVTxK, were performed within Excel itself. Transmembrane helix (TMH) prediction was performed at the Phobius server (https://phobius.sbc.su.se/). TMH clusters were determined in Excel. If more than one TMH was present within 100 amino acid of another, they were deemed to belong to a TMH cluster. A TMH architecture was defined by the number of clusters and number of TMHs in each cluster. All sequences were analysed for the presence of PFAM domains at the Motif server (https://www.genome.jp/tools/motif/). All PFAM domains identified were placed in one of five main domain groups, GT2, ZB (zinc binding), pilZ, GH (glycosyl hydrolase), and other. A domain group architecture was defined by the presence or absence of these domain groups in a given sequence. A domain architecture was also determined for each sequence, where the best domain (based on e-value and score) from each of the five groups was used.

### Phylogenetics

For producing the guide tree, 44 210 selected sequences were aligned using FAMSA (Deorowicz *et al*., 2016) and the guide tree inferred. For classification of CESA sequences, a smaller subset of 6580 sequences was aligned with MUSCLE (Edgar, 2004), and a maximum-likelihood tree was produced using FastTree (Price *et al*., 2010).

### Plant material

Secondary cell wall CESA T-DNA mutants, *cesa4^irx5–4^* (SALK_084627), *cesa7^irx3^*^–*7*^ (GABI_819B03), *cesa7^irx3^*^–6^ (SAIL_24_B10), and *cesa8^irx1^*^–*7*^ (GABI_339E12) were obtained from the NASC and homozygous plants were identified. Double and triple mutant combinations, *cesa4,7* (*cesa4^irx5–4^cesa7^irx3^*^–6^), *cesa4,8* (*cesa4^irx5–4^cesa8^irx1–7^*), *cesa7,8* (*cesa7^irx3^*^–6^*cesa8^irx1^*^–*7*^), and *cesa4,7,8* (*cesa4^irx5–4^cesa7^irx3^*^–6^*cesa8^irx1^*^–*7*^) were obtained by crossing the single mutants. All these mutants have been previously described in Kumar and Turner (2015).

### DNA cloning and plant transformation

Plasmids containing *Physcomitrium* CESA cDNA clones, pdp10281 (PpCESA3), pdp21409 (PpCESA4), pdp24095 (PpCESA5), pdp16421 (PpCesA6), pdp34523 (PpCESA7), and pdp39044 (PpCESA8) were obtained from the Riken Bioresource Research centre, Japan. These clones were used as template to re-clone the genes in a Gateway-compatible vector, pDONOR/pZeo, by adding attB adapter sequences in the cloning primers (Supplementary Table S1). When compared with the coding sequences (CDSs) from the Physcomitrium genome sequencing project, cloned PpCESAs contained some sequence differences. These included a 1 bp deletion and a splicing error in PpCESA3 (Pp3c8_7420V3.1), two substitution mutations, P490L and T858A, in PpCESA5 (Pp3c2_13330V3.1), two substitution mutations, D344G and R460G, in PpCESA6 (DQ902547.1), and two substitution mutations, K205E and P434S, in PpCESA7 (Pp3c15_7150V3.1). These sequences were ‘repaired’ using the overlap extension technique described previously (Atanassov *et al*., 2009; Kumar *et al*., 2017, 2018). The repair primers are listed in Supplementary Table S1. *Selaginella moellendorffii* total RNA was prepared from leaf midrib tissue using the Aurum RNA extraction kit (Bio-Rad Laboratories), and double-stranded cDNA was made with the iScript cDNA synthesis kit (Bio-Rad Laboratories). The cDNA was then used to amplify *Selaginella* CESAs, SmCESA2 (EFJ38258.1) and SmCESA4 (EFJ38300.1), which were then cloned into pDONOR/pZeo. CDS fragments for SmCESA1 (EFJ35421.1), SmCESA3 (EFJ37830.1), all four algal CESAs ACESA_MICDE (ADE44904.1, *Micrasterias denticulata*), ACESA_SPISP (HAOX-2004235, *Spirogyra* sp.), ACESA_CYLCU (JOJQ-2007927, *Cylindrocystis cushleckae*), and ACESA_MESEN (WDCW-2003349, *Mesotaenium endlicherianum*), and the fern CESA UOMY-2008646 from *Osmunda* were all synthesized commercially by BioBasic, Canada. During synthesis an additional two amino acids were added to ACESA_MICDE based on sequence alignment with other algal CESA proteins. These sequences were codon optimized for Arabidopsis and included the attB adapter-flanking sequences to facilitate Gateway cloning. All sequences were cloned into pDONOR/pZEO and verified using Sanger sequencing. Once validated, all sequences were transferred to a Gateway destination vector VX83 (pAtCESA7::GW, hygromycin plant selection, based on pCB1300 backbone). To make the double CESA plasmids, a complete expression cassette for PpCESA8 (pCESA7::PpCESA8:tNOS) was excised and ligated into the *Asc*I site of the plasmids containing PpCESA6 and PpCESA7. Sequenced plasmids were transformed into the *Agrobacterium* strain, pGV3101, which were then transformed into Arabidopsis plants using the floral dip method (Clough and Bent, 1998).

### Plant growth and analysis

T_1_ seeds were harvested from dipped Arabidopsis plants and selected on 1/2 Murashige and Skoog (MS) plates containing 35 µg ml^–1^ hygromycin. After growing for 7 d on plates in an incubator, 8–10 independent lines for each construct were transplanted into a 1:1:5 mixture of perlite, vermiculite, and compost. Plants were grown for a further 8 weeks on soil under long-day conditions (16 h/8 h day/night, 22 °C/18 °C temperature, and 80% humidity). The Col-0 wild-type (WT) and the *cesa* mutants were grown on plates without any selection before being transplanted. Vector-only controls for Col-0 WT and *cesa* mutants were included in two of the five experiments, and no differences were found in the growth patterns or cellulose content compared with corresponding genotypes grown on non-selection plates. Plant height measurements were taken when plants were 7 weeks old, after which 50 mm pieces from the primary inflorescence stem, starting at 5 mm above the base, were harvested and stored in 70% ethanol to analyse the cellulose content as described (Kumar and Turner, 2015). T_2_ seeds were collected from the secondary inflorescences that were left intact.

Plant height (cm) and cellulose content (% cell wall) were converted into plant height (% complementation) and cellulose content (% complementation) to assess the level of complementation. The data were imported into the IBM SPSS statistics program for statistical analysis. Univariate ANOVA with a least significant difference (LSD) post-hoc test was used to calculate the significance levels for the differences in the means.

### Solid-state NMR

Solid-state NMR analysis was performed as described previously (Kumar *et al*., 2018). For each genotype, 27 T_2_ plants were grown on plates and compost, as described above. Stem material was harvested when plants were 9 weeks old and stripped of their leaves and siliques. Stems were freeze-dried for 72 h and ground into a fine powder using a bead mill (TissueLyser II from Qiagen, UK). The alcohol-insoluble residue (AIR) was prepared by extracting twice with 70% ethanol (at 70 °C) and once with a 1:1 mixture of chloroform:methanol. Samples were then de-starched using amylase and pullulanase.

Solid-state NMR analysed up to 50 mg of de-starched AIR powder in cross-polarization/magic-angle spinning (CP/MAS) experiments. Carbon-13 MAS measurements were carried out at 100.63 MHz using a Bruker Avance III HD spectrometer and 4 mm (rotor o.d.) probe. Spectra were acquired at a spin rate of 10 kHz. CP spectra were recorded with TOSS spinning sideband suppression, 1 ms contact time, and a recycle delay of 2 s. Carbon spectral referencing is relative to neat tetramethylsilane, carried out by setting the high-frequency signal from an external sample of adamantane to 38.5 ppm. Recorded spectra were exported to Microsoft Excel, where they were normalized to make the total signal for each spectrum 100%. Further plotting of the spectra was performed in Excel.

### Digestibility assays

Enzymatic saccharification analysis was performed using the automated system as previously described (Gomez *et al*., 2010). Samples were processed in 96-well plates. For each genotype, up to 24 replicates (6 biological×4 technical), each weighing 4 mg, were analysed. Enzymatic saccharification and determinations of reducing sugars were performed in a liquid handling robotic platform (Tecan Evo 200; Tecan Group Ltd. Männedorf, Switzerland), with the enzyme cocktail Cellic® Ctec3 (7 FPU g^–1^) at 50 °C in 25 mmol l^–1^ sodium acetate buffer pH 4.5 for 18 h. Automated determination of the reducing sugars released after hydrolysis was performed by colorimetric assay using 3-methyl-2-benzothiazolinone hydrazone (MBTH). Data were expressed as sugar release (nmol mg^–1^ of AIR).

### Cell wall monosaccharide analysis

To analyse the monosaccharide content of non-cellulosic polysaccharides, cell wall material was hydrolysed with 2 M trifluoroacetic acid (TFA) for 4 h at 100 °C before separation by high-performance anion-exchange chromatography (HPAEC) on a CarboPac PA-20 column with pulsed amperometric detection (PAD). The HPAEC analysis was performed in a Dionex (Thermo) ICS-6000 PAD system with an electrochemical gold electrode. After equilibration in water, the monosaccharides were separated in a 5 min gradient from 0 to 2 mM NaOH. The following 10 min were run at 2 mM NaOH before a gradient of 7 min to reach 45 mM NaOH in 30% solution of 500 mM NaOAc/100 mM NaOH buffer, maintaining these conditions for 15 min. The flow was kept constant at 0.5 ml min^–1^. The separated monosaccharides were quantified by external calibration using an equimolar mixture of monosaccharide standards, which had also been treated with 2 M TFA in the same way as the samples.

## Results

### Phylogeny of CESA proteins

**Fig. 1.**
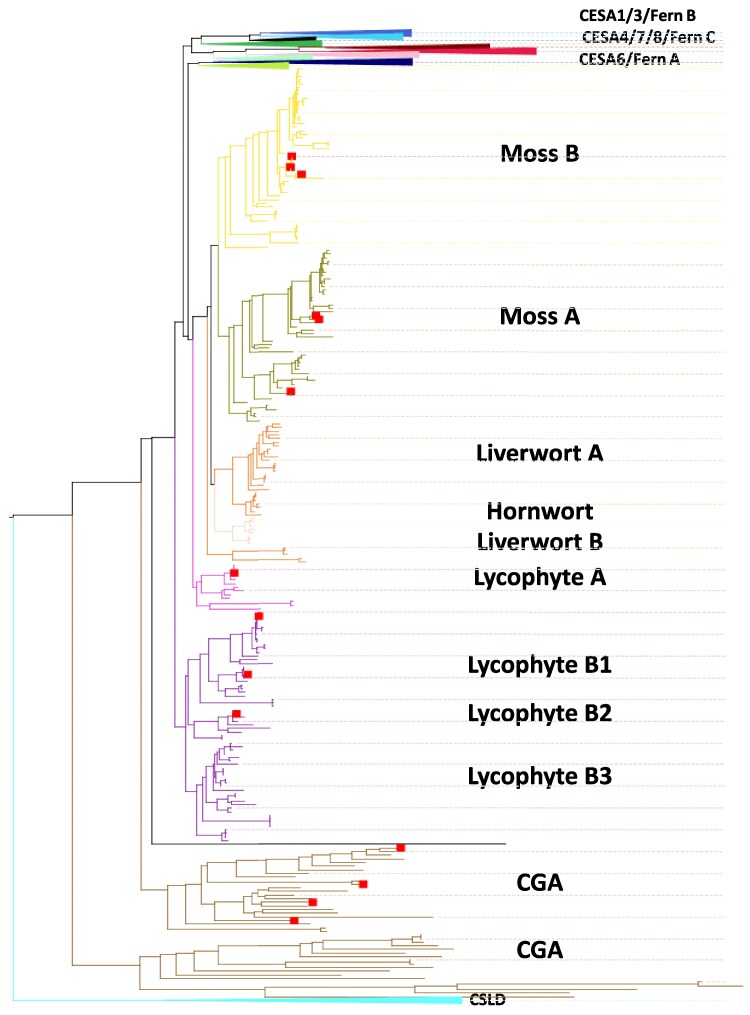
Phylogenetic tree showing the classification of Streptophyta CESA and CSLD sequences. A selection of 6580 Streptophyta sequences containing the GT2 domain and the RING domain in their N-terminus were aligned with MUSCLE, and a maximum likelihood (ML) tree was inferred using Fast-tree. Six phylogenetic classes of CESA proteins from seed plants are labelled based on their Arabidopsis members. The tree is organized to highlight the details of bryophyte, lycophyte, and CGA sequences. The 15 proteins used for complementation studies are indicated by squares (the square for fern class C is hidden as that part of the tree has been collapsed to highlight the details of bryophyte, lycophyte, and CGA CESAs). An alternative representation of the same tree is shown in Supplementary Fig. S1.

We analysed a large number of manually curated CESA and cellulose synthase-like D (CSLD) sequences from across the taxonomic diversity of Streptophyta. A total of 6580 plant CESA and CSLD sequences that possessed the typical features of rosette-type CESA proteins were aligned and used to generate a phylogenetic tree (Fig. 1; Supplementary Fig. S1). There was a clear separation of CESA and CSLD sequences. CESA sequences from seed plants formed six separate clades for the six well-established CESA phylogenetic classes named after the Arabidopsis members CESA1, CESA3, and CESA6 that are involved in primary cell wall biosynthesis, and CESA4, CESA7, and CESA8 required for secondary cell wall synthesis (Fig. 1; Supplementary Fig. S1). We also identified three CESA phylogenetic classes in ferns, Fern A, Fern B, and Fern C, positioned with CESA6, CESA1/3, and CESA4/7/8 classes from angiosperms, respectively. These results are comparable with those obtained by Pancaldi *et al*. (2022), who performed the most comprehensive phylogenetic analysis of CESA sequences from seed plants published to date.

In contrast, the CESA sequences from bryophytes, lycophytes, and CGA species do not mix with sequences from seed plants and ferns or each other, forming separate clades for each group (Fig. 1; Supplementary Fig. S1). However, when multiple CESA sequences are present in these species, they separate into ‘phylogenetic classes’. There appeared to be at least two distinct classes for each taxonomic group (class A and class B). For lycophytes, class B is split into three further subgroups (Fig. 1). However, the functional distinctness is only established for the moss *Physcomitrium patens*, which has two distinct CESA classes, with a member from each class being required for cellulose synthesis (Norris *et al*., 2017; Scavuzzo-Duggan *et al*., 2018; Li *et al*., 2019). Within a phylogenetic tree of CESA proteins from green plants, these moss CESA proteins form a separate clade from seed plant CESAs (Fig. 1).

### Functional analysis of CESA proteins from a diverse range of green plants

To test whether CESA proteins were functionally equivalent to Arabidopsis secondary cell wall CESA proteins, we expressed 15 CESAs selected from a broad range of green plants into the Arabidopsis *cesa4*, *cesa7*, and *cesa8* single and higher order mutants. The selected proteins covered a range of taxonomic diversity (Fig. 1) and comprised one CESA protein from each of four different CGA species, six CESA proteins from the moss *P. patens*, all four CESA proteins from spike moss *S. moellendorffii*, and a single CESA protein from the fern *Osmunda* that came from group C, the clade that appears to have most recently shared a common origin with the secondary cell wall CESA proteins (Fig. 1; Supplementary Fig. S1; acession numbers given in Supplementary Table S2). Despite the evolutionary distance, the large regions and the overall structure of the proteins are well conserved (Supplementary Fig. S2).

We chose to study the complementation of Arabidopsis mutants caused by mutations in genes encoding CESA4, CESA7, and CESA8, which are all essential for cellulose synthesis in the secondary cell walls. None of the *cesa4*, *cesa7*, or *cesa8* single mutants, any combination of double mutants, or the *cesa4,7,8* triple mutant can synthesize cellulose in the secondary cell wall (Kumar and Turner, 2015; Kumar *et al*., 2017, 2018). Despite this cellulose deficiency, plants are still viable, and previous studies have demonstrated that the cellulose-deficient phenotype of all *cesa4*, *cesa7*, and *cesa8* single mutants as well as all double mutant combinations and the triple mutant may be complemented by expressing the appropriate cDNA clones (Kumar *et al*., 2017, 2018).

Genes corresponding to the selected 15 proteins, expressed using the Arabidopsis CESA7 promoter, were transformed into each Arabidopsis single secondary cell wall mutant, *ces4*, *cesa7*, or *cesa8*, as well as all double mutant combinations and the triple mutant, leading to the generation of nearly 100 genotypes. The rosette area, plant height, and cellulose content were measured for complementation analysis. All genotypes were analysed at the T_1_ stage, while a selection of genotypes was also analysed again in the T_2_ generation. As a result of the large number of genotypes involved, plants were grown across multiple batches with the WT and *cesa* mutant controls included with each batch (Supplementary Fig. S3). All data are expressed as the percentage complementation (Kumar *et al*., 2018) to facilitate comparisons between different batches. In this calculation, the WT is 100% and the *cesa* mutant is 0%. We measured both cellulose content and plant height as we have previously observed a strong correlation between these two traits. Still, in some instances, small increases in cellulose content led to more significant increases in plant height (Kumar *et al*., 2018). An overview of the data is summarized in Table 1.

**Table 1.** Summary of complementation data

Class	CESA or species	Cellulose content (% complementation)							Plant height (% complementation)						
Algal	*M. denticulata*	ND	ND	ND	NA	16	4	NA	−1	6	0	NA	6	1	−1
	*Spirogyra* sp.	ND	ND	ND	−2	−4	0	NA	4	4	0	4	6	1	4
	*C. cushleckae*	ND	ND	ND	−7	2	4	NA	8	−1	4	1	6	6	−1
	*M. endlicherianum*	NA	ND	ND	NA	22	−4	NA	NA	0	−2	NA	6	−7	−3
Fern C	*Osmunda* sp.	−7	1	−3	NA	−1	NA	1	4	−13	−2	NA	9	NA	2
Lycophyte	SmCESA1	3	4	4	7	4	7	8	−2	0	0	1	1	1	−2
	SmCESA2	−8	−11	−15	2	3	2	−14	−29	−26	−24	−6	8	1	−44
	SmCESA3	7	6	1	NA	NA	NA	NA	−5	−4	−7	NA	NA	NA	NA
	SmCESA4	**18**	6	−5	0	2	1	−10	**66**	24	−21	0	2	10	−33
Moss A	PpCESA3	2	−15	−12	0	1	11	−11	5	−35	−25	2	8	12	−17
	PpCESA5	−5	−6	−1	1	3	2	4	−5	−37	−18	5	2	7	−19
	PpCESA8	**28**	−6	−6	−1	2	−2	−5	**116**	−3	−4	4	13	−13	−12
Moss B	PpCESA4	−10	12	−11	1	0	2	−7	−31	**80**	−35	6	0	7	−63
	PpCESA6	−9	**52**	0	**44**	4	**44**	−1	−6	**103**	**16**	**48**	9	**64**	−13
	PpCESA7	−2	**33**	−11	3	1	**29**	−8	18	**81**	19	9	10	**44**	−14
Moss A+B	PpCESA6+8	NA	NA	NA	NA	NA	NA	**16**	NA	NA	NA	NA	NA	NA	**33**
	PpCESA7+8	NA	NA	NA	NA	NA	NA	**19**	NA	NA	NA	NA	NA	NA	**22**
		*cesa4*	*cesa7*	*cesa8*	*cesa4,7*	*cesa4,8*	*cesa7,8*	*cesa4,7,8*	*cesa4*	*cesa7*	*cesa8*	*cesa4,7*	*cesa4,8*	*cesa7,8*	*cesa4,7,8*

A total of 15 CESA proteins were expressed in 7 mutant backgrounds (bottom row), where one or more of the Arabidopsis secondary cell wall CESAs was knocked out. Complementation data for cellulose content and plant height is shown. Significant complementation is indicated by bold type. NA, no lines available; ND, cellulose content not determined.

**Fig. 2.**
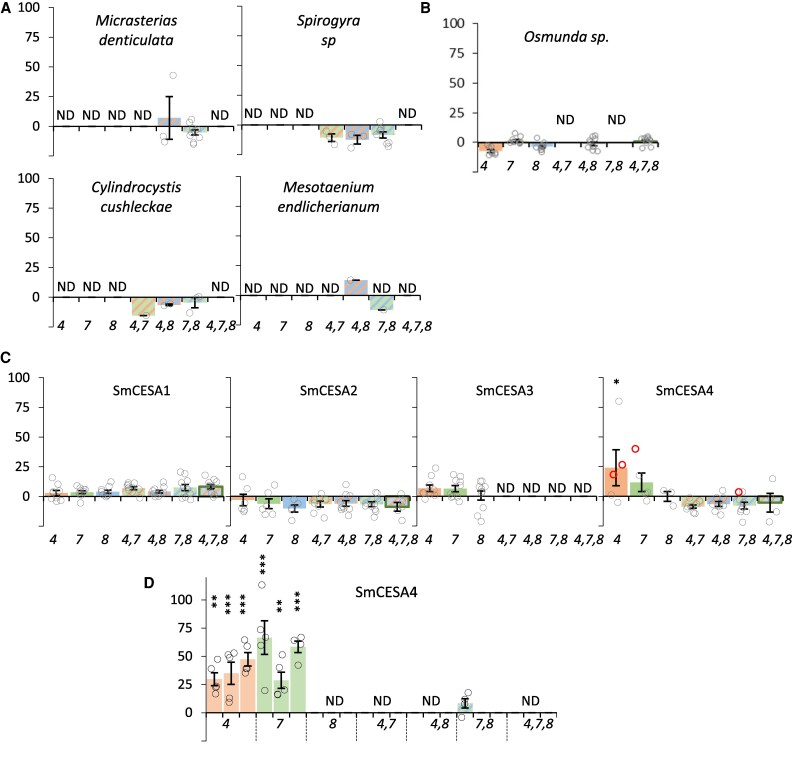
Complementation of the cellulose-deficient phenotype of Arabidopsis secondary cell wall CESA mutants expressing CESA from four different species of CGA, the fern *Osmunda*, and the spikemoss, *Selaginella*. Genes encoding single CESA proteins were expressed in Arabidopsis *cesa4,7,8* single and multiple mutant knockout combinations. (A) Genes encoding CESA proteins from four species of CGAs. (B) A gene encoding a CESA protein from the fern *Osmunda* species, a member of fern class C. (C) Genes encoding four different CESA proteins from *Selaginella moellendorffii*. (D) T_2_ generation of SmCESA4. Results are expressed as percentage complementation. *cesa* mutants are indicated by the number in italics on the *x*-axis. Up to 10 individual T_1_ plants were measured for each genotype, and mean values are shown with SE bars. Open circles indicate individual measurements, and the lines selected for T_2_ analysis are marked with bold red circles. Significantly positive complementation, calculated using univariate ANOVA between the genotype and the cesa4,7,8 triple mutant, is indicated with asterisks. ***Significant at 0.001, **significant at 0.01, *significant at 0.05. ND – not determined.

None of the CESA sequences from the four CGA species could complement the plant height or cellulose content defects of the mutants (Table 1; Fig. 2A; Supplementary Fig. S4A). These results indicate that these CESA proteins can form neither functional complexes with any of the Arabidopsis CESA proteins nor functional homomeric complexes by themselves in Arabidopsis.

Since fern class C appears related to seed plant secondary cell wall CESA proteins but appears to have diverged before the diversification of the secondary cell wall CESA proteins into different classes (Fig. 1; Supplementary Fig. S1), we hypothesized that this fern group might form homomeric rosettes. To test this hypothesis, we analysed the ability of a class C CESA protein from the fern *Osmunda* to complement the *cesa4*, *cesa7*, and *cesa8* mutants. As with the CGA genes, we observed no complementation using this fern gene (Fig. 2B; Supplementary Fig. S4B; Table 1).

We also tested all four CESA proteins from *Selaginella*, a lycophyte, for their ability to complement Arabidopsis *cesa4*, *cesa7*, and *cesa8* mutants (Fig. 2C; Supplementary Fig. S4C). Out of the four CESA genes, only SmCESA4 exhibited any significant complementation. It could complement both the cellulose content and plant height defects of *cesa4*. While the complementation of *cesa7* was not significant overall in the T_1_ generation, three lines appeared to exhibit good complementation. The complementation of *cesa4* and *cesa7* was verified in the T_2_ generation (Fig. 2D; Supplementary Fig. S4D). None of the *Selaginella* genes could complement the cellulose content of the Arabidopsis *cesa* higher order mutants (Fig. 2C; Supplementary Fig. S4C). However, we did obtain one line for SmCESA4, which appeared to show slight complementation of the plant height phenotype of the *cesa7,8* double mutant (Fig. 2C; Supplementary Fig. S4C). Comparable results were obtained when we tested this line in the T_2_ generation, with significant complementation of plant height phenotype but not the cellulose content.

**Fig. 3.**
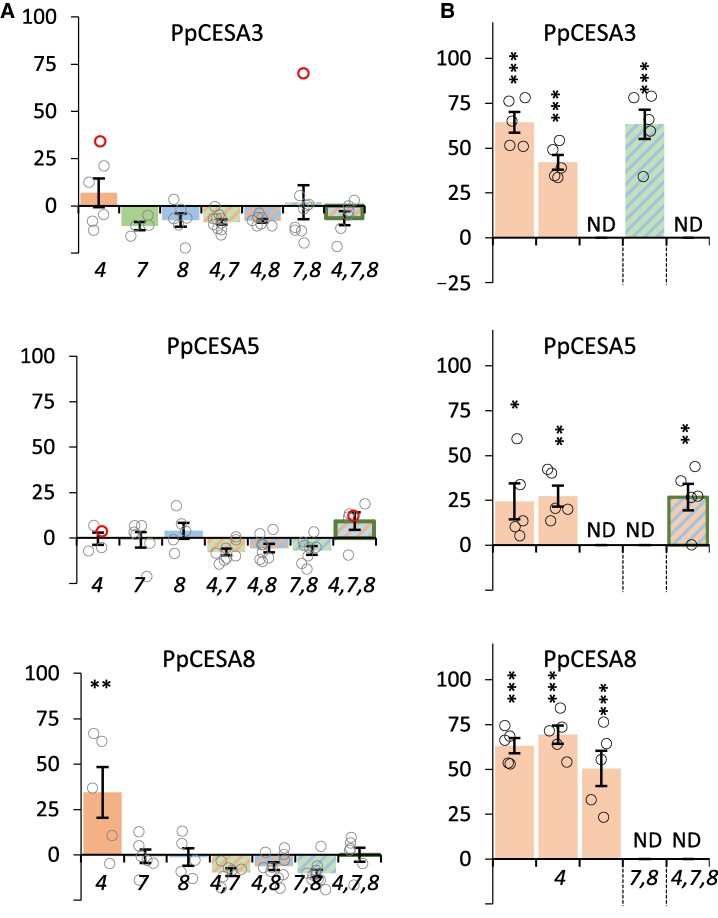
Complementation of the cellulose-deficient phenotype of Arabidopsis secondary cell wall *cesa* mutants expressing CESA proteins from the moss, *Physcomitrium patens* class A. Individual CESA proteins were expressed in Arabidopsis *cesa4, cesa7, cesa8* single and multiple mutant knockout combination mutants. *cesa* mutants are indicated by the number in italics on the *x*-axis. Results are expressed as percentage complementation. (A) T_1_ measurements. (B) T_2_ measurements. Individual T_1_ plants were measured for each genotype, and mean values are shown with SE bars. Open circles represent all individual measurements, and the lines selected for T_2_ analysis are indicated with a bold red circle. For T_2_ analysis, up to three independent lines were grown, and five plants were measured for each line. Significantly positive complementation, calculated using univariate ANOVA between the genotype and the *cesa4,7,8* triple mutant, is indicated with asterisks. *** Significant at 0.001, ** significant at 0.01, * significant at 0.05. ND, not determined.

Genetic analysis of the moss *P. patens* has previously demonstrated that CESA genes fall into two distinct phylogenetic and functional classes (Norris *et al*., 2017; Tran *et al*., 2018; Li *et al*., 2019). Moss class A includes PpCESA3, 5, and 8, while class B includes PpCESA4, 6, 7, and 10 (Scavuzzo-Duggan *et al*., 2018). CESAs from both classes are involved in cellulose synthesis in primary cell walls (PpCESA5, 4, and 10) and secondary cell walls (PpCESA3, 8, 6, and 7) (Norris *et al*., 2017; Li *et al*., 2019). Among the moss class A CESAs, PpCESA8 was able to partially, but significantly, complement both the cellulose-deficient and plant height defect of the *cesa4* mutant (Fig. 3A; Supplementary Fig. S5A; Table 1). Additionally, some individual complemented T_1_ lines were obtained when PpCESA3 and PpCESA5 were expressed in the *cesa4* mutant, although the results were not statistically significant when tested across 10 independent T_1_ lines (Fig. 3A). Moss class A CESAs did not complement the other single, double, or triple *cesa* mutants (Fig. 3A; Table 1), except one individual line each for the *cesa7,8* double mutant expressing PpCESA3 and the *cesa4,7,8* triple mutant expressing PpCESA5 (Fig. 3; Supplementary Fig. S5). To verify these data, we selected up to three independent lines from each genotype that exhibited at least some level of complementation and analysed the T_2_ generation. The selections were based mainly on plant height complementation in the T_1_ generation (red circles in Supplementary Fig. S5A). The results were broadly similar to those obtained in the T_1_ generation, with all three class A CESA genes from moss able to complement *cesa4* and form a functional complex together with the Arabidopsis CESA7 and CESA8 proteins (Fig. 3B; Supplementary Fig. S5B).

**Fig. 4.**
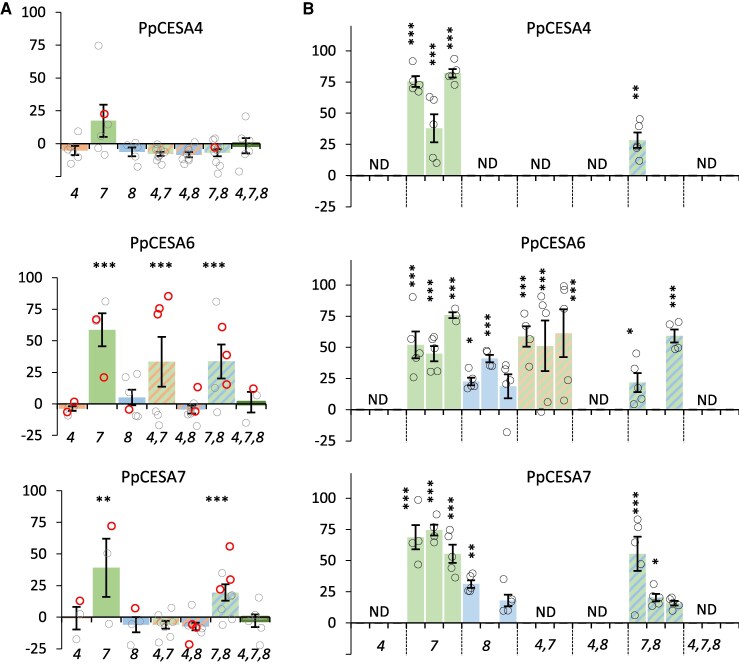
Complementation of the cellulose-deficient phenotype of Arabidopsis secondary cell wall *cesa* mutants expressing CESA proteins from the moss, *Physcomitrium patens* class B. Individual CESA proteins were expressed in Arabidopsis *cesa4, cesa7, cesa8* single and multiple mutant knockout combination mutants. *cesa* mutants are indicated by the number in italics on the *x*-axis. Results are expressed as percentage complementation. (A) T_1_ measurements. (B) T_2_ measurements. Individual T_1_ plants were measured for each genotype, and mean values are shown with SE bars. Open circles represent all individual measurements, and the lines selected for T_2_ analysis are indicated with bold red circles. For T_2_ analysis, up to three independent lines were grown and five plants were measured for each line. Significantly positive complementation, calculated using univariate ANOVA between the genotype and the *cesa4,7,8* triple mutant, is indicated with asterisks. *** Significant at 0.001, ** significant at 0.01, * significant at 0.05. ND, not determined.

When we analysed complementation following expression of the moss class B CESAs, PpCESA4, 6, and 7, we were able to obtain significant complementation of the cellulose content of the *cesa7* mutant with both PpCESA6 and PpCESA7 proteins (Fig. 4A; Table 1) while all three class B *Physcomitrium* genes were able to complement the plant height phenotype of *cesa7* (Supplementary Fig. S6A). Furthermore, both PpCESA6 and PpCESA7 were also able to complement the *cesa8* mutant, albeit to a lesser extent than the *cesa7* mutant, with only the complementation observed with PpCESA6 being statistically significant (Fig. 3B; Table 1). None of the moss class B CESAs was able to complement the *cesa4* mutant. The results were verified in the T_2_ generation, with all three moss class B genes able to complement the *cesa7* mutant (Fig. 4B; Supplementary Fig. S6B). Complementation with PpCESA6 was particularly effective as it was able to complement the plant height and cellulose defect of the *cesa4,7* and *cesa7,8* double mutants in addition to complementing both the cellulose and plant height defects of *cesa7* and the plant height defect of *cesa8* single mutants (Fig. 4; Supplementary Fig. S6).

The fact that multiple CESA proteins are required to make cellulose in green plants complicates the engineering of CESA protein structure to alter cellulose synthesis. PpCESA5 is unique among the *Physcomitrium* CESA proteins for its ability to synthesize cellulose without other CESA proteins in both the primary cell wall buds of the protonema and the secondary cell wall of the leaf midribs (Li *et al*., 2022). This ability makes PpCESA5 the most likely candidate to complement the Arabidopsis *cesa4,7,8* triple mutant. Most lines expressing PpCESA5 in the triple *cesa* mutant exhibited no complementation in the T_1_ generation. One line that showed the clearest complementation of the plant heigh defect (Supplementary Fig. S5) also demonstrated significant complementation of the cellulose defect in the T_2_ generation (Fig. 3B).

### Moss CESA proteins alone are sufficient to synthesize cellulose in Arabidopsis

**Fig. 5.**
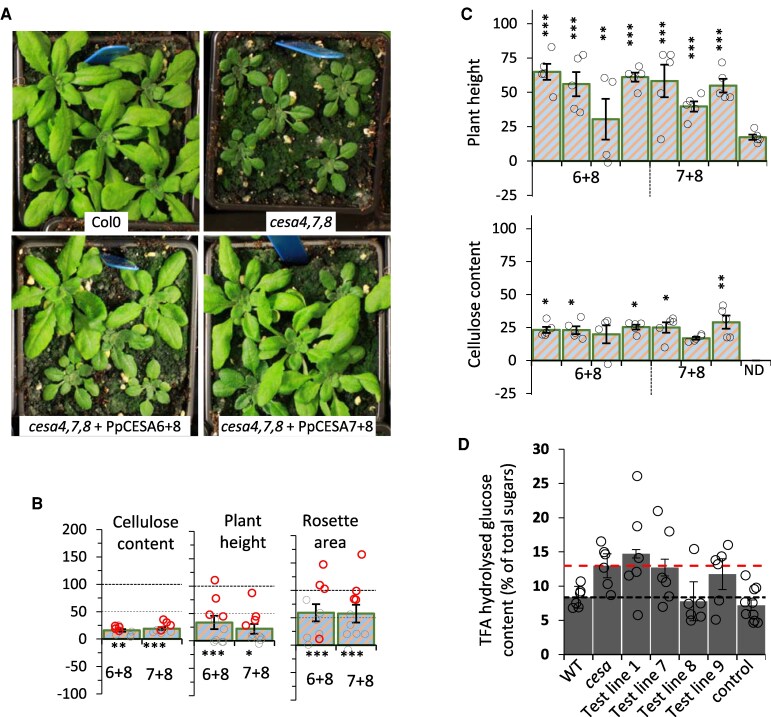
Complementation of the Arabidopsis *cesa4,7,8* triple mutant by expressing two *Physcomitrium* CESA proteins. PpCESA6 and PpCESA8 (6+8) or PpCESA7 and PpCESA8 (7+8) were expressed in the Arabidopsis *cesa4,7,8* triple mutant. (A) Images of representative plants at 4 weeks old. (B) Complementation of stem cellulose content, plant height, and rosette area. Ten individual T_1_ plants were measured for each genotype. Open circles represent all individual measurements, and the lines selected for T_2_ analysis are indicated with red circles. (C) Complementation of cellulose content and plant height phenotype of selected T_2_ lines. Significantly positive complementation, calculated using univariate ANOVA between the genotype and the *cesa4,7,8* triple mutant, is indicated with asterisks. *** Significant at 0.001, ** significant at 0.01, * significant at 0.05. (D) Total sugars were released by hydrolysis with 2 M TFA, measured using HPAEC analysis on a Dionex (Thermo) ICS-6000 PAD system, and expressed as a percentage of total sugars. Data for glucose content are shown here, while all nine sugars measured are shown in Supplementary Fig. S7. Test lines refer to four independent lines of *cesa4,7,8* expressing PpCESA6+PpCESA8. These are the same lines as in (C). ‘Control’ refers to a group of four different genotypes—PpCESA6 transformed into the Arabidopsis *cesa7* mutant; PpCESA6 transformed into the Arabidopsis *cesa4,7* mutant; PpCESA6 transformed into the Arabidopsis *cesa7,8* mutant, and PpCESA8 transformed into the Arabidopsis *cesa4* mutant. These lines were chosen because they complemented both the plant height and cellulose defect of the mutants equally well (Figs 3B, 4B; Supplementary Figs S5B, S6B).

Our experiments with expression of single moss CESAs showed that class B moss CESAs, PpCESA6 and PpCESA7, were both able to complement the *cesa7* mutant and also the *cesa8* mutant to a lesser extent, but not the *cesa4* mutant, although we did obtain T_2_ plant height complementation for a single line each for both PpCESA6 and PpCESA7. In contrast, PpCESA8, a class A moss CESA, effectively complemented the *cesa4* mutant. These data are consistent with a previous genetic analysis in moss, which demonstrated that members from both class A and B were required to synthesize cellulose (Li *et al*., 2019). With that in mind, we created two plasmids containing either PpCESA6 and PpCESA8 or PpCESA7 and PpCESA8 and expressed them in the *cesa4,7,8* triple mutant background. Both combinations were able to significantly complement the triple mutant for plant height, rosette area, and cellulose content phenotypes (Fig. 5), indicating that the pairs of *Physcomitrium* proteins were able to synthesize cellulose in the secondary cell wall independently of any Arabidopsis genes.

While we observed significant complementation of cellulose content in these plants overexpressing two *Physcomitrium* CESA genes, we observed far more substantial changes in plant height and rosette area, with some of the complemented lines growing as well as the WT (Fig. 5A, B). We have previously performed complementation analyses similar to those presented here and observed that the plant height phenotype is a more sensitive indicator of complementation, where relatively small increases in cellulose content can lead to far more significant increases in plant height (Kumar *et al*., 2017, 2018). However, the discrepancy between cellulose content and plant height for the triple mutant complemented with two moss CESAs was more substantial than anything we have previously observed (Kumar *et al*., 2017, 2018) or with the other genotypes presented in this study. We selected four independent lines for each genotype to study this result further. The results confirmed those obtained for the T_1_ plants, with all T_2_ lines exhibiting good complementation of plant height but much smaller, albeit significant, complementation of the defect in cellulose content (Fig. 5C).

**Fig. 6.**
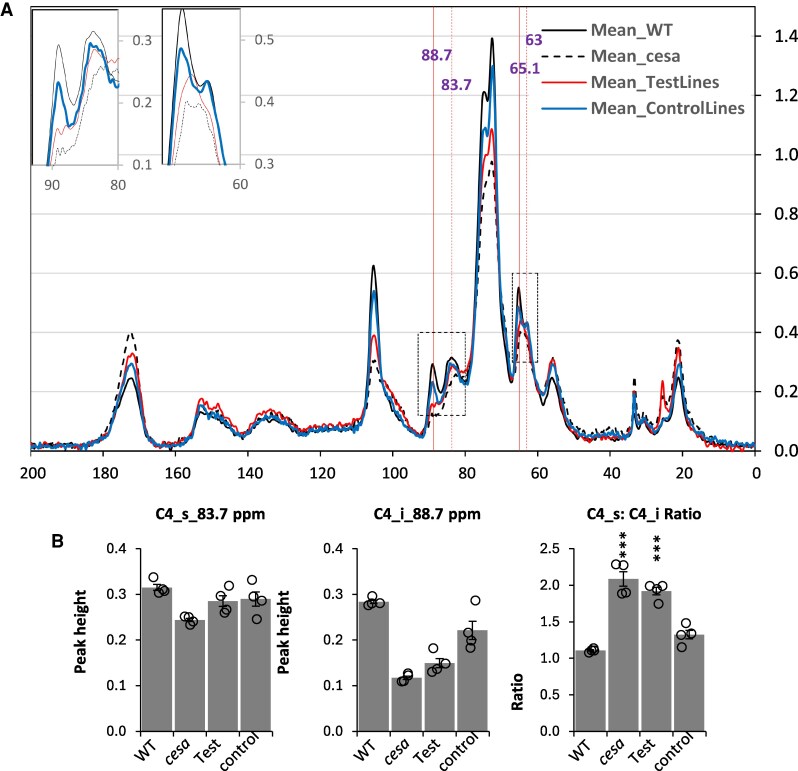
Solid-state NMR analysis of the Arabidopsis *cesa4,7,8* triple mutant complemented with *Physcomitrium* genes PpCESA6 and PpCESA8. (A) Traces are the means of spectra obtained from four different samples. ‘TestLines’ is the mean of four independent lines of the *cesa4,7,8* triple mutant transformed with PpCESA6 and PpCESA8, while the ‘Control lines’ are the mean of four different genotypes—PpCESA6 transformed into the Arabidopsis *cesa7* mutant; PpCESA6 transformed into the Arabidopsis *cesa4,7* mutant; PpCESA6 transformed into the Arabidopsis *cesa7,8* mutant, and PpCESA8 transformed into the Arabidopsis *cesa4* mutant. Vertical lines indicate positions for peaks associated with interior and surface cellulose (C4_i_88.7 ppm, C4_s_83.7 ppm, C6_i_65.1 ppm, C6_s_63 ppm). (B) Peak heights for all samples were extracted at wave numbers 88.7 ppm and 83.7 ppm, and surface to interior cellulose ratios were calculated. Four replicates for each genotype are shown along with means. Error bars are the SEM. Significant increases in the C4_s:C4_i ratio compared with the WT are indicated with asterisks. *** Significant at 0.001.

All our cellulose content measurements are performed using a variation of Updegraf’s method that only measures crystalline cellulose (Updegraff, 1969). Solid-state NMR can detect peaks for interior cellulose chains (indicative of crystalline cellulose) and surface cellulose chains (indicative of amorphous cellulose) (Dupree *et al*., 2015). To obtain a more precise overview of changes in cell wall composition of these lines, we used solid-state NMR to analyse Col-0 WT, the *cesa4,7*,*8* triple mutant, and four independent lines for the *cesa4,7,8* triple mutant complemented with *PpCESA6* and *PpCESA8*. As controls, we also analysed genotypes from this study that exhibited good complementation for both plant height and cellulose content, and included one line for each PpCESA6 transformed into the *cesa7* single and the *cesa4,7* and *cesa7,8* double mutants, and a line with PpCESA8 transformed into the *cesa4* mutant (Fig. 6). We paid particular attention to the pair of peaks at 88.7 ppm and 83.7 ppm associated with C4 and those at 65.1 ppm and 63 ppm associated with C6 that correspond to the interior and surface chains of cellulose, respectively. Comparison of the signal from WT samples with those from the *cesa4,7,8* triple mutant clearly shows significant differences in peaks corresponding to cellulose, including the four peaks listed above (Fig. 6). For the control lines, with increasing cellulose content, the signal for both interior and exterior chains increased proportionately to generate a spectrum that more closely resembles the WT (Fig. 6). In contrast, for the lines expressing the *Physcomitrium* proteins PpCESA6 and PpCESA8, there is a significant increase in the signal for exterior cellulose peaks at 83.7 ppm and 63 ppm. Still, the increase in signal from the interior chains at 88.7 ppm and 65.1 ppm was significantly smaller (Fig. 6). This result indicates the presence of increased non-crystalline or amorphous cellulose in these lines. We then performed monosaccharide sugar analysis (Fig. 5D; Supplementary Fig. S7) on the same material to investigate if an increased surface/interior cellulose ratio was correlated with increased soluble sugar content. Indeed, both the *cesa* mutant and the *cesa* mutant complemented with PpCESA6+PpCESA8, which had a high surface/interior cellulose ratio (Fig. 6B), also had high glucose content (Fig. 5D).

### Improved saccharification in lines synthesizing cellulose co-expressing PpCESA6 and PpCESA8

**Fig. 7.**
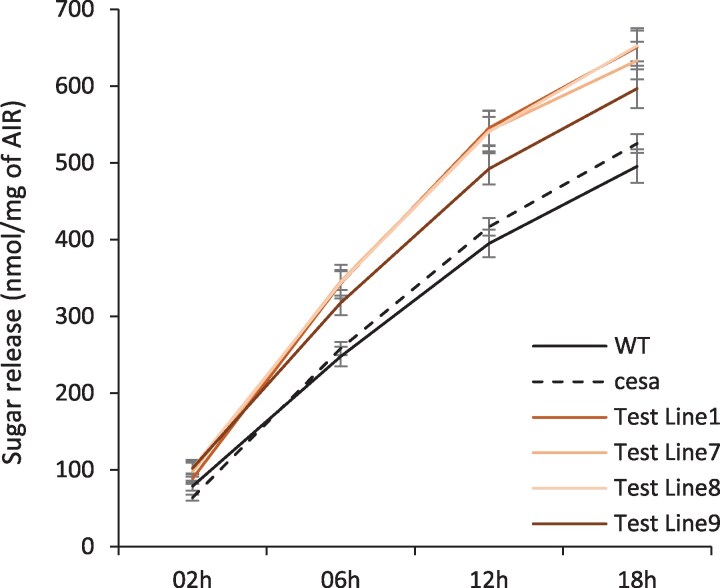
Digestibility assays of the Arabidopsis *cesa4,7,8* triple mutant complemented with *Physcomitrium* genes PpCESA6 and PpCESA8. Sugar release data, without any pre-treatment for four independent lines of PpCESA6 and PpCESA8, transformed into the *cesa4,7,8* triple mutant, are shown. Each data point is a mean of 24 replicates (6 biological×4 technical). Error bars are the SEM. Sugar release from all four lines is significantly more at 6, 12, and 18 h with *P*-value <0.05.

We performed a digestibility assay on these samples to test whether the increased proportion of non-crystalline cellulose we observed in plants overexpressing two *Physcomitrium* genes might have any practical applications. In a saccharification assay measuring sugar release from samples without any pre-treatment, we observed a significant increase in sugar release using samples from four independent lines expressing both *PpCESA6* and *PpCESA8* in the *cesa4,7,8* triple mutant compared with either the WT or the *cesa4,7,8* triple mutant controls (Fig. 7). This would support the idea that cellulases more easily digest the less crystalline cellulose generated by the PpCESA6 and PpCESA8 proteins. Notably, these plants grow normally and can achieve 100% plant height complementation in some cases (Fig. 5B), meaning that there is little or no growth penalty involved. These plants provide an example of how cellulose synthesis could be manipulated to produce an improved feedstock for biobased chemicals and fuels.

## Discussion

Phylogenetic analysis suggests that CGA sequences show little evidence of gene duplication and diversification, and it is likely that *in situ*, most CGA species can form homomeric rosette structures. Despite the conservation of their overall CESA structure and large regions of high sequence conservation, these CESAs from the CGAs appear to be sufficiently diverse to be unable to synthesize cellulose in Arabidopsis secondary cell walls, even in the presence of two of the three Arabidopsis CESA proteins required. While the proteins appear structurally conserved, it is clear that CESA proteins in Arabidopsis require extensive post-translation modification (Chen *et al*., 2010; Kumar *et al*., 2016; Mergner *et al*., 2020) and the CESA proteins from CGA species may not be modified appropriately. Similarly, based on the phylogenetic data, we hypothesize that fern class C CESA proteins will be likely to form a homomeric complex. Still, the single CESA gene we tested from this group failed to complement *cesa* mutants, potentially because the fern and angiosperm secondary cell wall CESA proteins have diverged considerably from their common ancestor (Supplementary Fig. S1). Among the 15 CESA sequences tested, all possess identical sequences at the four main catalytic motifs: DD (DDG), DxD (DCD), TED (TED), and QxxRW (QVLRW), and the gating loop FxxVxK (FTVTS/AK) (Supplementary Fig. S2). Availability of the crystal structure of a homo-trimer of heterologously expressed PttCESA8 revealed 25 contact points between different subunits, located mainly in the P-CR, TM4, and TM7 regions (Purushotham *et al*., 2020). However, all these points are identical in nearly all Arabidopsis CESAs and the 15 CESAs tested here (Supplementary Fig. S2). Most differences between Arabidopsis and those CESA sequences from green plants other than angiosperms reside within highly variable regions close to the N-terminus (VR1) and the central cytoplasmic domain (VR2, which covers most of the region known as the class-specific region or CSR) (Supplementary Fig. S2). The structure of these regions is poorly understood and both contain known sites for post-translational modifications, including phosphorylation (Chen *et al*., 2010; Mergner *et al*., 2020) and *S*-acylation (Kumar *et al*., 2016), as well as interacting with other proteins. As these regions are so variable between different CESA proteins, it is plausible that their failure to complement a particular *cesa* mutant or to function independently during Arabidopsis secondary cell wall biosynthesis may result from either lack of or alterations in essential post-translational modifications, or their inability to interact with other proteins necessary for cellulose synthesis.

We deliberately avoided adding any epitope tags to the CESA proteins tested in this study to ensure that the tags themselves did not interfere with the structure and complementation of the protein. Consequently, one caveat when interpreting the lack of complementation is the lack of data regarding the expression levels of the proteins in Arabidopsis. Genes for seven out of 15 CESA proteins tested in this study (four CGA CESs, one fern CESA, and two *Selaginella* CESAs) were synthesized and codon optimized for expression in Arabidopsis. Furthermore, we have previously expressed many chimeric and mutant CESA proteins in Arabidopsis (Kumar *et al*., 2016, 2017, 2018, 2022). The vast majority of these chimeric and mutant CESA proteins exhibited reasonable expression levels, even for the lines that showed little or no complementation, and there appeared to be no significant correlation between levels of complementation and protein expression (Kumar *et al*., 2017, 2018). Consequently, while we consider lack of expression unlikely to cause a particular CESA protein not to exhibit complementation of any mutants, we cannot rule out this possibility, and negative results must be interpreted cautiously. This point is less relevant for the CESA proteins from *Physcomitrium* as all the CESA proteins we tested exhibited some degree of complementation consistent with significant levels of expression (Figs 3, 4; Supplementary Figs S5, S6).

**Fig. 8.**
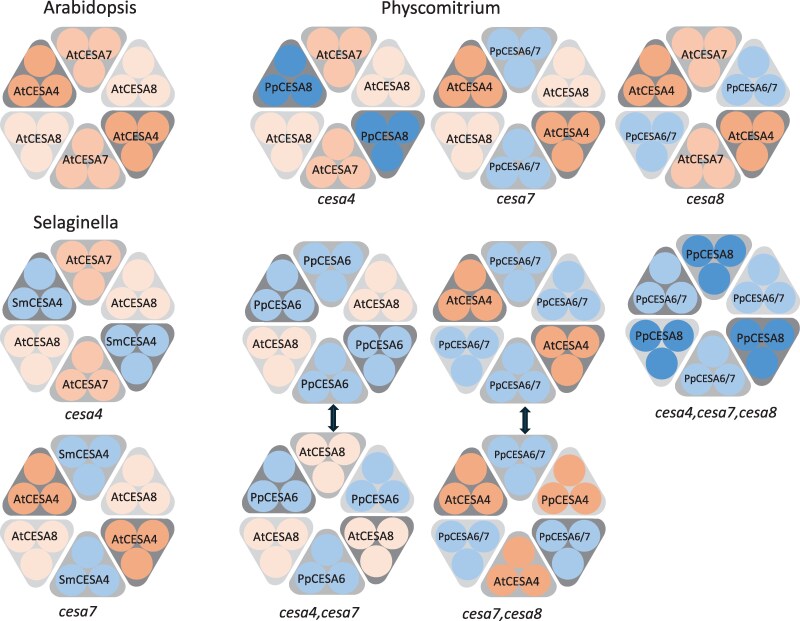
Diagrammatic representation of CESA protein organization in WT Arabidopsis and *cesa* mutant combinations complemented with heterologously expressed CESA genes. The Arabidopsis organization assumes that the complex is an 18-mer composed of six homotrimers. Two alternative organizations are presented for the *cesa4,7* and *cesa7,8* complementation with PpCESA6 and/or PpCESA7 that cannot be distinguished based on complementation data alone.

A summary of the potential organization of CESA proteins within an 18-mer composed of six lobes based upon the complementation data is shown in Fig. 8. In contrast to the CGA CESA, the CESA proteins from the moss *Physcomitrium* can function in Arabidopsis. Notably, the two functional classes of CESA proteins identified in *Physcomitrium* are required to synthesize cellulose in Arabidopsis. A combination of the class A protein PpCESA8 and the class B genes PpCESA6 or PpCESA7 can complement the triple mutant and synthesize cellulose independently of any Arabidopsis CESA proteins. These data provide independent support for genetic studies in *Physcomitrium*, demonstrating that a CESA from class A and B is required to make cellulose (Norris *et al*., 2017; Li *et al*., 2019).

Although crystallinity is one of the fundamental properties of cellulose, precisely what determines the proportion of ordered versus amorphous cellulose is unclear. Mutations in Arabidopsis CESA proteins can reduce the crystallinity of the cellulose synthesized (Harris *et al*., 2012). However, this does not address what determines the differences in structural properties between the cellulose synthesized in primary cell walls and that synthesized in secondary cell walls or other examples of natural variation in cellulose structure. The cellulose synthesized by the moss CESA proteins in Arabidopsis was less crystalline than that synthesized in the secondary cell walls of the WT (Fig. 6). These alterations in the ratio of surface to interior cellulose are more significant than those observed when we mutated the catalytic aspartates in CESA4, CESA7, or CESA8 (Kumar *et al*., 2018), a change that we would anticipate would reduce the size of a microfibril by a third. This suggests that the changes observed in the cellulose produced by moss PpCESA8 with either PpCESA6 or PpCESA7 are not a consequence of simply altering the number of chains within a microfibril. While these proteins generate cellulose with more surface-exposed chains, their cellulose appears to be sufficiently structurally robust to allow substantial complementation of the plant growth defect that is so apparent in Arabidopsis secondary cell wall mutants (Kumar *et al*., 2017, 2018). While the exact cause of the alteration in cellulose structure is unclear, it is unlikely to be the result of the local cellular environment as the cellulose synthesized by the *Physcomitrium* CESA proteins is identical to that in which the Arabidopsis CESA proteins function. Consequently, the properties of the cellulose synthesized are likely to be a result either of the structure of the CESA proteins themselves or of changes in protein–protein interactions. This includes interactions between the *Physcomitrium* CESA proteins and other Arabidopsis proteins known to function in cellulose biosynthesis, such as the putative endoglucanase KORRIGAN, that also affect cellulose microfibril structure (Takahashi *et al*., 2009).

The fact that moss class B CESA proteins can complement *cesa7* and *cesa8* mutants but not *cesa4* (Fig. 8) is at odds with the phylogenetic data that indicate that CESA7 diverged before the divergence of the CESA4/CESA8 clade, resulting in the sequences of CESA4 and CESA8 being significantly more similar to one another than they are to CESA7. Furthermore, functional analyses of chimeric secondary cell wall CESA proteins involving domain swaps from other secondary cell wall CESAs show that CESA7 exhibits little functional complementation when domains from either CESA4 or CESA8 are swapped in, suggesting that CESA7 has stringent structural constraints (Kumar *et al*., 2017). The basis of this apparent promiscuity revealed for the structure of moss class B CESAs is hard to understand without reliable structural data. While the structural information for the conserved catalytic domains of CESA proteins is available (Purushotham *et al*., 2020), the diverse regions likely to be responsible for the class specificity, such as the CSR region, are currently lacking. Our domain swap experiments (Kumar *et al*., 2017) have demonstrated that many regions of the proteins contribute to class specificity. While the current study does not specifically identify which regions of the proteins define the class specificity, it is likely that known variable regions such as the VR1 and CSR (which includes VR2) that exhibit a high degree of intrinsic disorder are likely to be important (Scavuzzo-Duggan *et al*., 2018; Olek *et al*., 2023). Once more information about these regions becomes available, it should reveal important particulars of the fundamental rules governing CESA protein structure and assembly of the CSC. PpCESA5 appeared unique in its ability to partially complement the *cesa4,7,8* triple mutant, even though only one out of 10 lines showed complementation. Altering only a single CESA protein would significantly simplify CESA protein engineering to change their activity and the properties of the cellulose they produce. The partial complementation of the PpCESA5 gene represents a baseline that offers the opportunity to understand how we can increase the ability of this protein to fully complement or exceed the typical Arabidopsis secondary cell wall cellulose content by altering either expression levels or post-translational modification, or making other structural changes.

One of the challenges in reducing greenhouse gas emissions is to use renewable materials. The sheer abundance of cellulose means it is one of the few renewable feedstocks with the potential to make a significant impact on reducing greenhouse gas emissions. The utility of cellulose as a feedstock could be increased by generating a greater diversity of cellulose from abundant sources such as seed plants. For example, many red algae make a diverse array of much larger microfibrils (Tsekos, 1999). If they could be synthesized in seed plants, it would offer enormous potential for synthesizing new material or acting as novel feedstocks. This is the first report showing that CESA proteins from seed plants can be completely replaced with those from a moss species. By replacing CESA proteins from Arabidopsis with those from *Physcomitrium*, we demonstrate that it is possible to generate low recalcitrance cellulose with altered properties. This represents an important first step in developing new and improved materials and feedstocks from plant biomass.

## Supplementary Material

eraf511_Supplementary_Data

## Data Availability

All data supporting the findings of this study are available within the article and its supplementary data. Any further information can be obtained from the corresponding author upon reasonable request. All materials generated in this study will be made available upon request, for non-commercial purposes. The accession numbers for Arabidopsis genes mentioned in this paper are AT5G44030 (CESA4), AT5G17420 (CESA7), and AT4G18780 (CESA8). Supplementary Table S2 provides the accession numbers for the 15 heterologous proteins used in this study.

## Supplementary data

The following supplementary data are available at *JXB* online.

Fig. S1. Phylogenetic tree showing classification of Streptophyta CESA and CSLD sequences.

Fig. S2. An alignment of protein sequences for six Arabidopsis CESAs and the 15 CESAs tested in this study.

Fig. S3. Plant height and cellulose content measurements for the control genotypes.

Fig. S4. Complementation of the plant height phenotype of Arabidopsis secondary cell wall CESA mutants expressing CESA proteins from four different species of CGA, the fern *Osmunda*, and the spikemoss, *Selaginella*.

Fig. S5. Complementation of the plant height phenotype of Arabidopsis secondary cell wall *cesa* mutants expressing CESA proteins from the moss, *Physcomitrium patens* class A.

Fig. S6. Complementation of the plant height phenotype of Arabidopsis secondary cell wall *cesa* mutants expressing CESA proteins from the moss, *Physcomitrium patens* class B.

Fig. S7. Monosaccharide analysis of the Arabidopsis *cesa4,7,8* triple mutant complemented with *Physcomitrium* genes PpCESA6 and PpCESA8.

Table S1. Primers used in this study.

Table S2. Accession numbers and sequence sources for the 15 CESA proteins used in this study.
