# Supplementary material for: Exploiting evolutionary diversity of cellulose synthase catalytic subunits to generate novel cellulose microfibril structure in Arabidopsis
Source: J Exp Bot. 2025 Nov 21;77(6):1598–614. doi: 10.1093/jxb/eraf511 (PMC13016765; doi:10.1093/jxb/eraf511)
Supplement: eraf511_Supplementary_Data [file eraf511_supplementary_data.pdf]

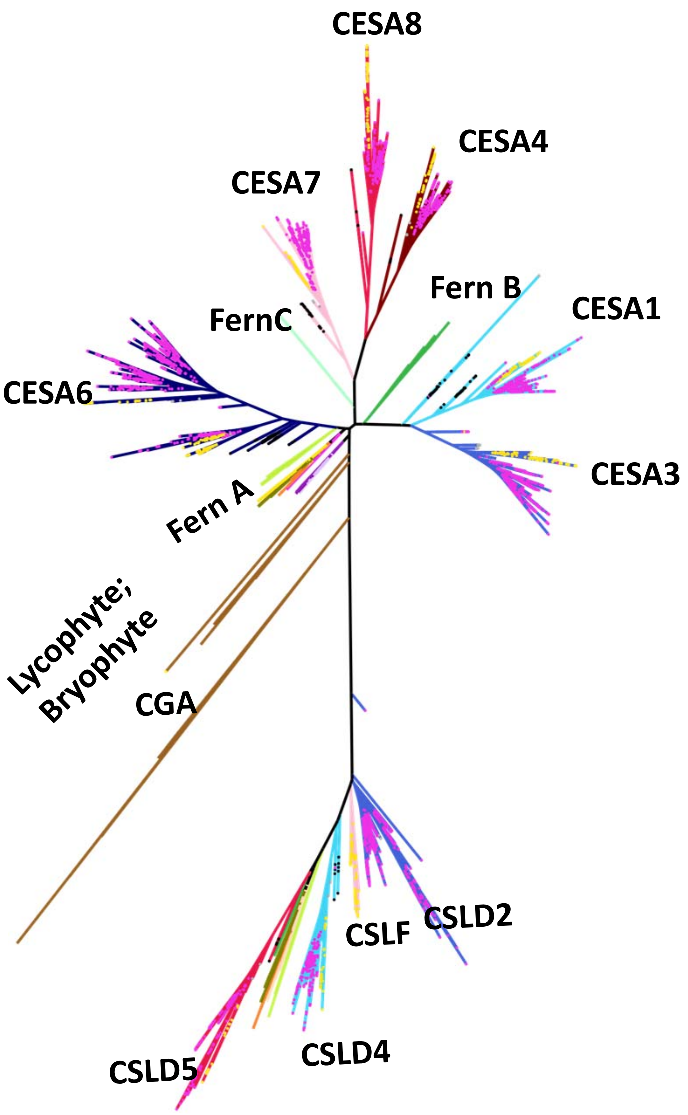

Supplementary Figure S1. Phylogenetic tree showing the classification of Streptophyta CESA and CSLD sequences.

A selection of 6580 Streptophyta sequences containing the GT2 and RING domains in their N-terminus were aligned with MUSCLE. A maximum likelihood (ML) tree was inferred using Fast-tree. Six phylogenetic classes of CESA proteins from seed plants are labelled based on their Arabidopsis members. Leaf dot colour indicates dicots (magenta), monocots (yellow) and gymnosperms (black). Fern CESA classes are well resolved (Fern A, B and C), while those from lycophytes, bryophytes (mosses, liverworts, hornworts) and CGA are clustered close together. This is an alternative representation of the same tree shown in Figure 1.

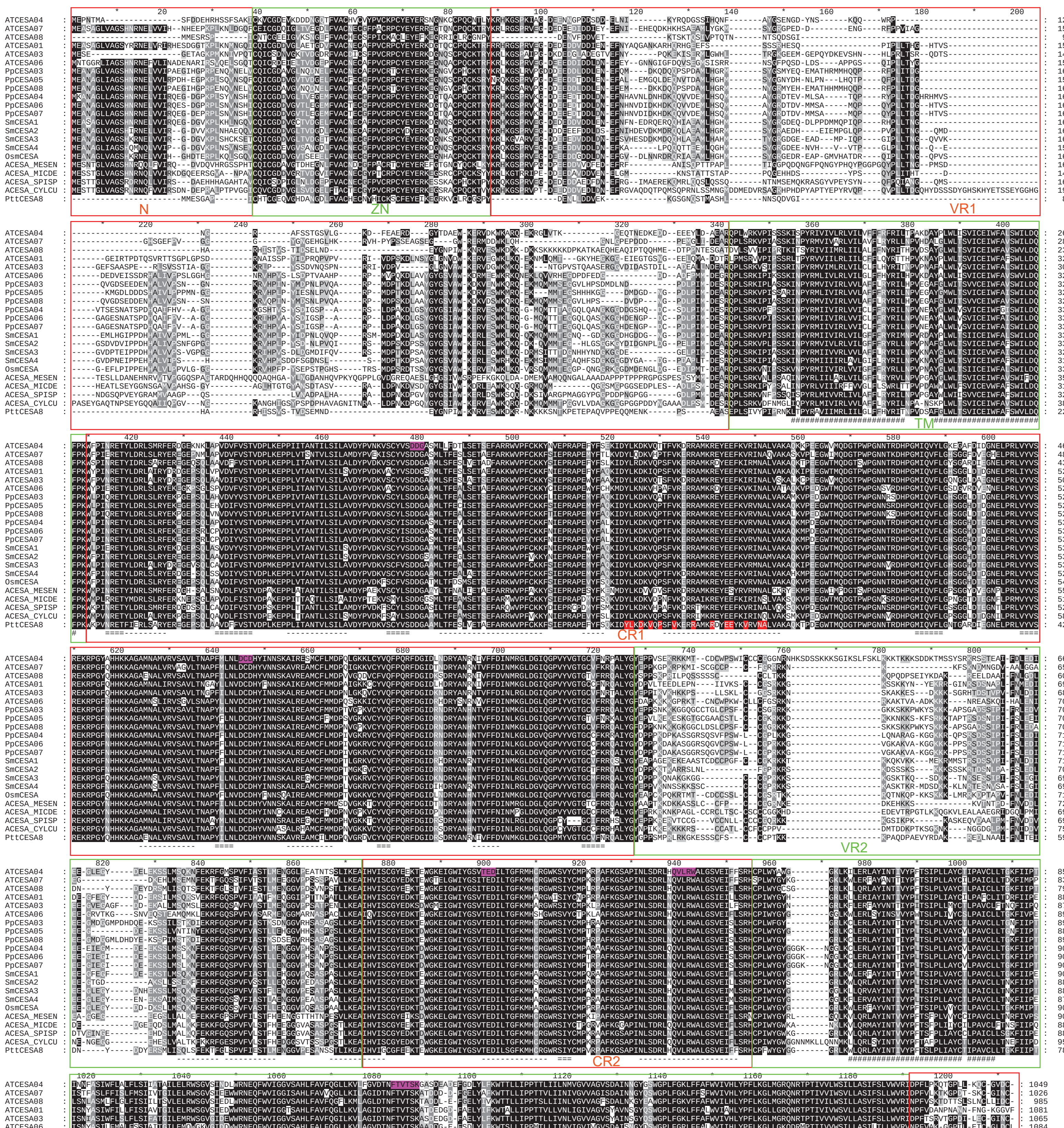

Supplementay Figure S2. An alignment of protein sequences for 6 Arabidopsis CESAs and the 15 lower plants CESAs tested in this study
PttCESA8, for a homo-trimeric structure has been published, is included to indicate position of various structural elements – TMH1-7 (indicated with # at the bottom of the alignment), interface helix (|), gating loop (:), alpha-helices (=), beta sheets (-). 25 amino acids involved in subunit contacts are shown with red shading on PttCESA8 sequence. Catalytically important motifs, DDG, DCD, TED, QVLRW and FTVTSK are highlighted in pink on ATCESA8 sequence. Approximate position of the 9 domains as described in Kumar et al, 2017 are shown with alternating red and green rectangles. The plant-conserved region (P-CR) is located within the CR1 region while the class specific region (CSR encompasses all of VR2 and the beginning of the CR2).

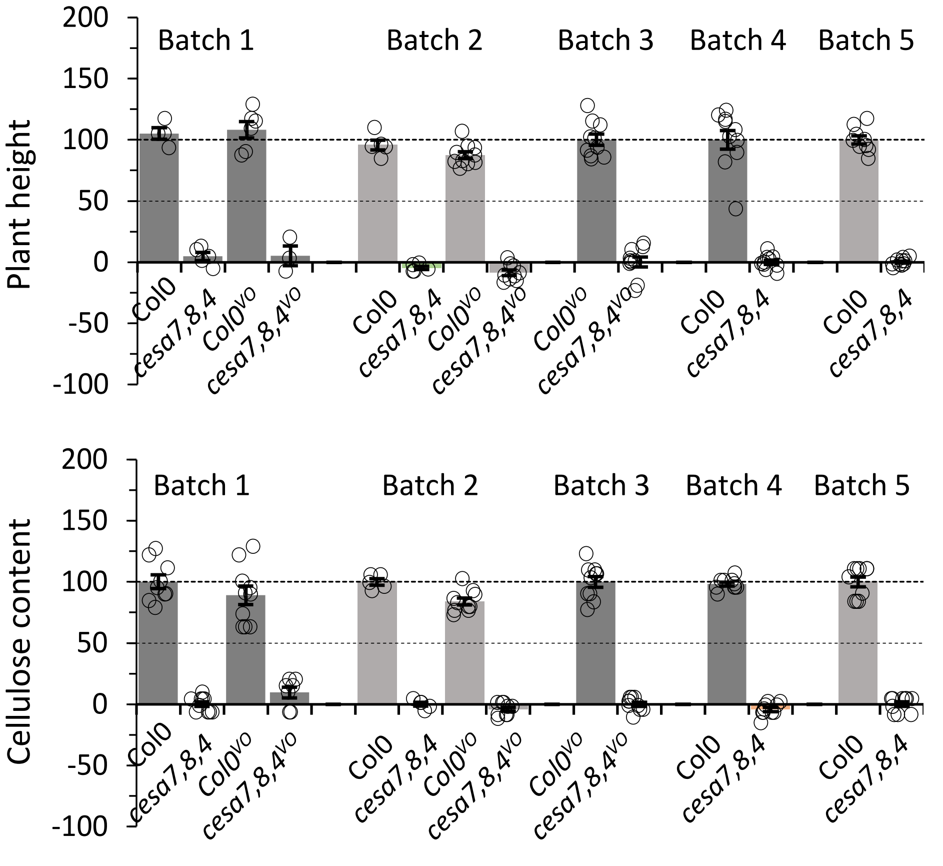

Supplementary Figure S3. Plant height and cellulose content measurements for the control genotypes

Plants were grown across five batches, and measurements were taken from 8-week-old plants. Wild-type and mutant control genotypes were grown in each batch, and the data was expressed as % complementation (refer to the method section for the calculation formula). *Col0^{VO}* and *cesa7,8,4^{VO}* refer to vector-only control transformed into Col0 and *cesa8 cesa7 cesa4* triple mutant, respectively. Empty circles represent all individual measurements. Significantly positive complementation, calculated using univariate ANOVA between the genotype and the cesa 7,8, 4 triple mutant, is indicated with asterisks. *** Significant at 0.001, ** significant at 0.01, * significant at 0.05.

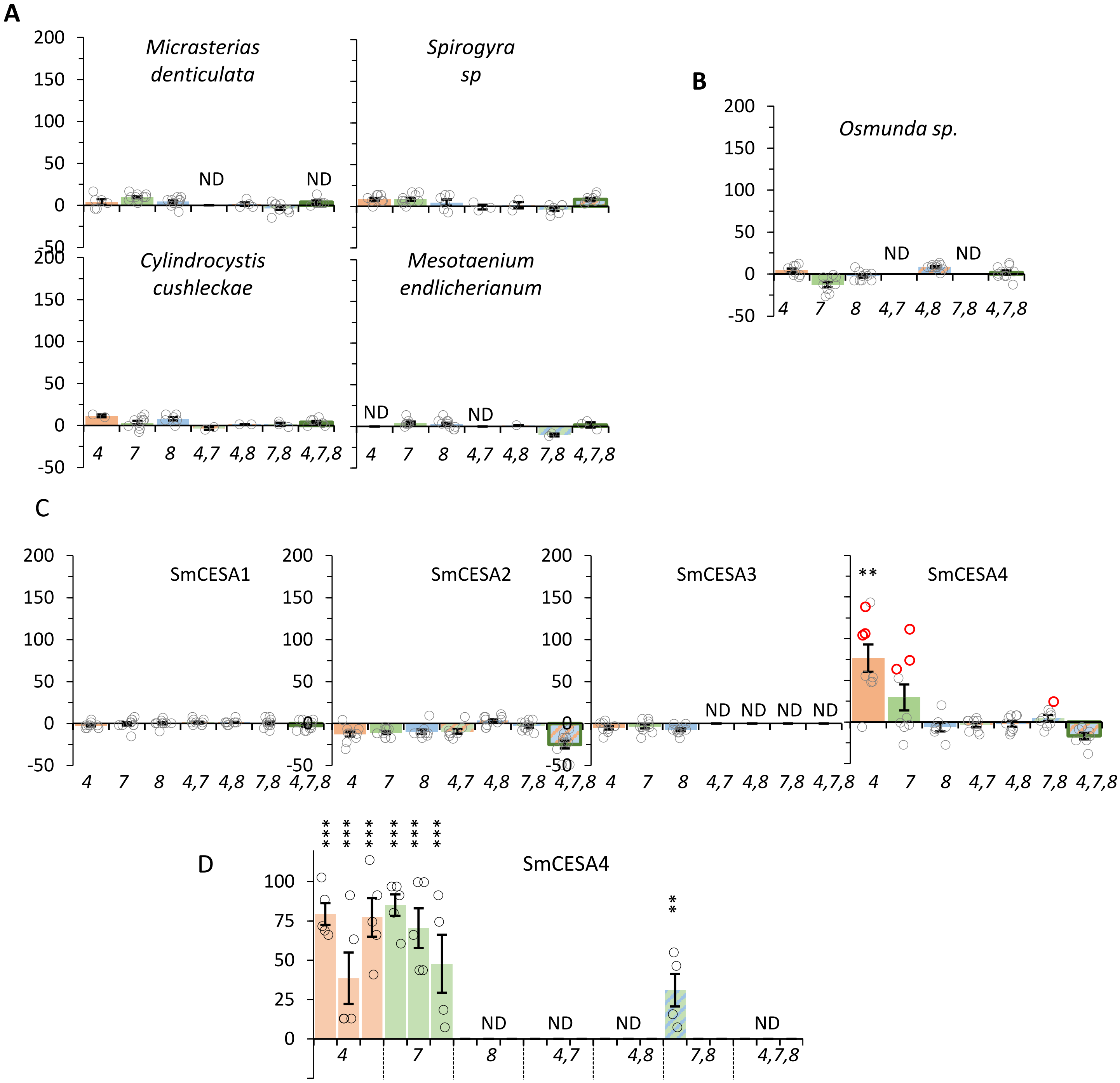

Supplementary Figure S4. Complementation of the plant height phenotype of Arabidopsis secondary cell wall CESA mutants expressing CESA proteins from 4 different species of CGA, the fern *Osmunda* and the spikemoss, Selaginella.

Genes encoding single CESA proteins were expressed in Arabidopsis *cesa4,7,8* single and multiple mutant knock-out combinations.

(A) Genes encoding CESA proteins from 4 species of CGAs.

(B) A gene encoding a CESA protein from fern *Osmunda species,* a member of fern group C.

(C) Genes encoding four different CESA proteins from *Selaginella moellendorffii*.

(D) T2 analysis of selected lines expressing SmCESA4

Results are expressed as % complementation. *Cesa* mutants are indicated by the number in italics on the x-axis. Individual T1 plants were measured for each genotype, and mean values are shown with standard error bars. Open circles indicate individual measurements, and the lines selected for T2 analysis are indicated with red circles. For T2 analysis, up to 3 independent lines were grown, and 4-5 plants were measured for each line. Significantly positive complementation, calculated using univariate ANOVA between the genotype and the *cesa4,7,8* triple mutant, is indicated with asterisks. *** Significant at 0.001, ** significant at 0.01, * significant at 0.05. ND – not determined.

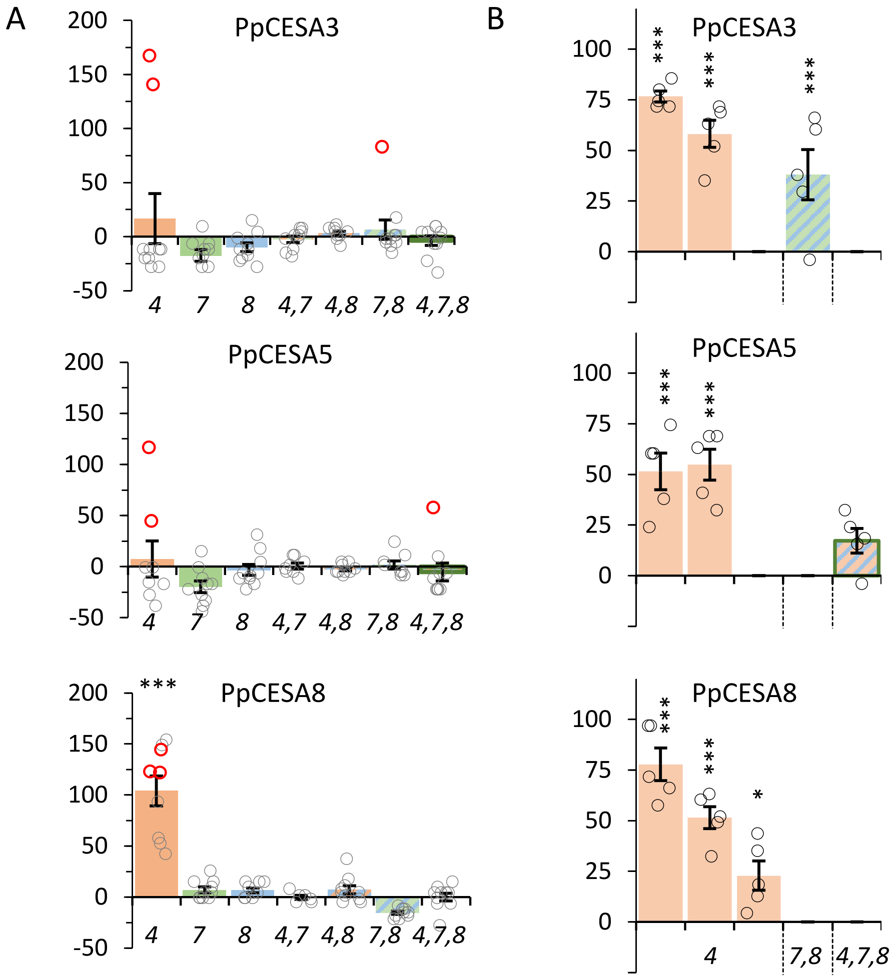

Supplementary Figure S5. Complementation of the plant height phenotype of Arabidopsis secondary cell wall *cesa* mutants expressing CESA proteins from the moss, *Physcomitrium patens* Class A.

Individual CESA proteins were expressed in Arabidopsis *cesa4,7,8* single and multiple mutant knock-out combination mutants. *Cesa* mutants are indicated by the number in italics on the x-axis. Results are expressed as % complementation.

(A) T1 measurements

(B) T2 measurements

Up to 10 individual T1 plants were measured for each genotype, and mean values are shown with standard error bars. Empty circles represent all individual measurements, and the lines selected for T2 analysis are indicated with red circles. For T2 analysis, up to 3 independent lines were grown, and five plants were measured for each line. Significantly positive complementation, calculated using univariate ANOVA between the genotype and the *cesa4,7,8* triple mutant, is indicated with asterisks. *** Significant at 0.001, ** significant at 0.01, * significant at 0.05. ND – not determined.

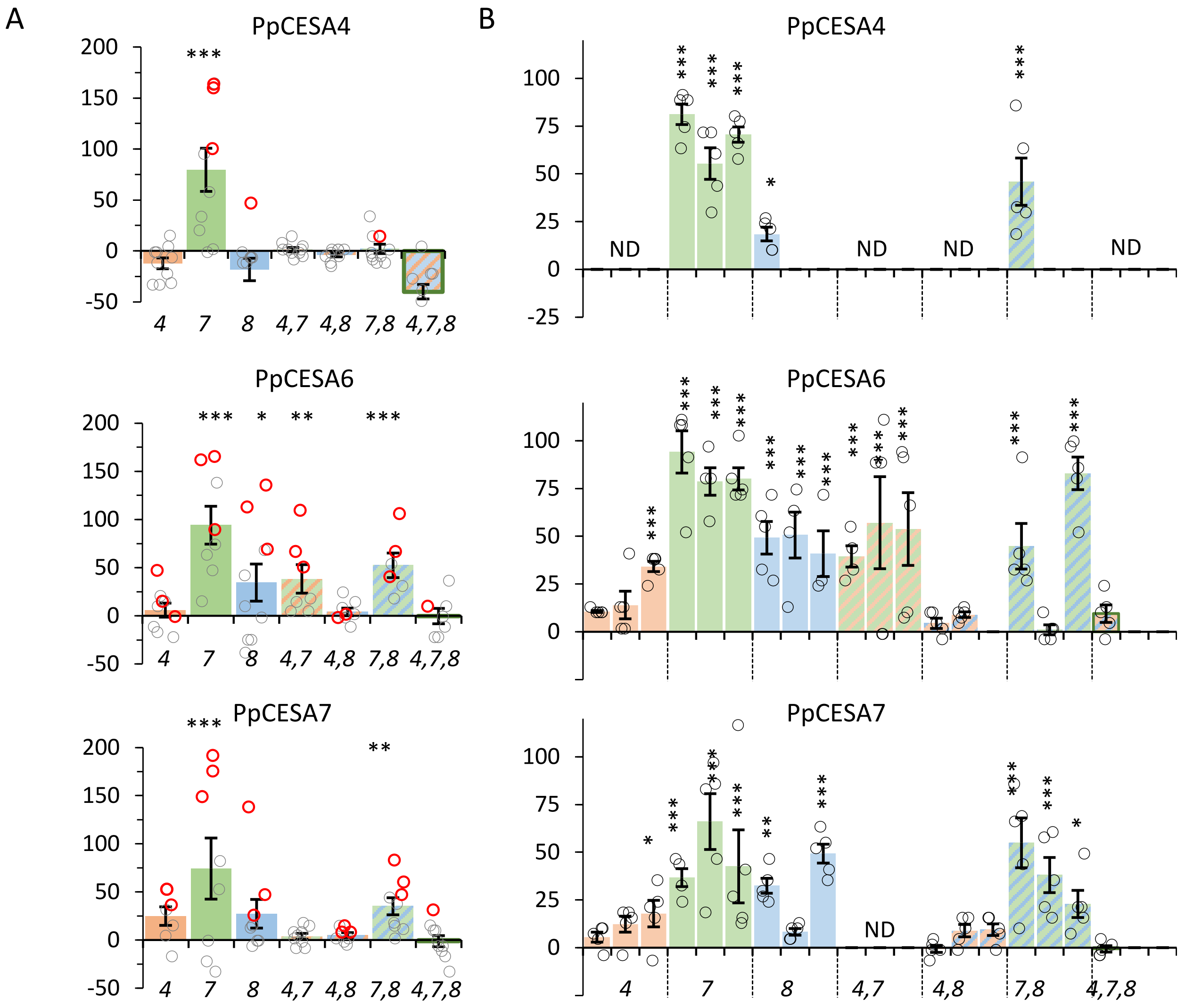

Supplementary Figure S6. Complementation of the plant height phenotype of Arabidopsis secondary cell wall *cesa* mutants expressing CESA proteins from the moss, *Physcomitrium patens* Class B.

Individual CESA proteins were expressed in Arabidopsis *cesa4,7,8* single and multiple mutant knock-out combination mutants. *Cesa* mutants are indicated by the number in italics on the x-axis. Results are expressed as % complementation.

(A) T1 measurements

(B) T2 measurements

Up to 10 individual T1 plants were measured for each genotype, and mean values are shown with standard error bars. Empty circles represent all individual measurements, and the lines selected for T2 analysis are indicated with red circles. For T2 analysis, up to 3 independent lines were grown, and five plants were measured for each line. Significantly positive complementation, calculated using univariate ANOVA between the genotype and the *cesa4,7,8* triple mutant, is indicated with asterisks. *** Significant at 0.001, ** significant at 0.01, * significant at 0.05. ND – not determined.

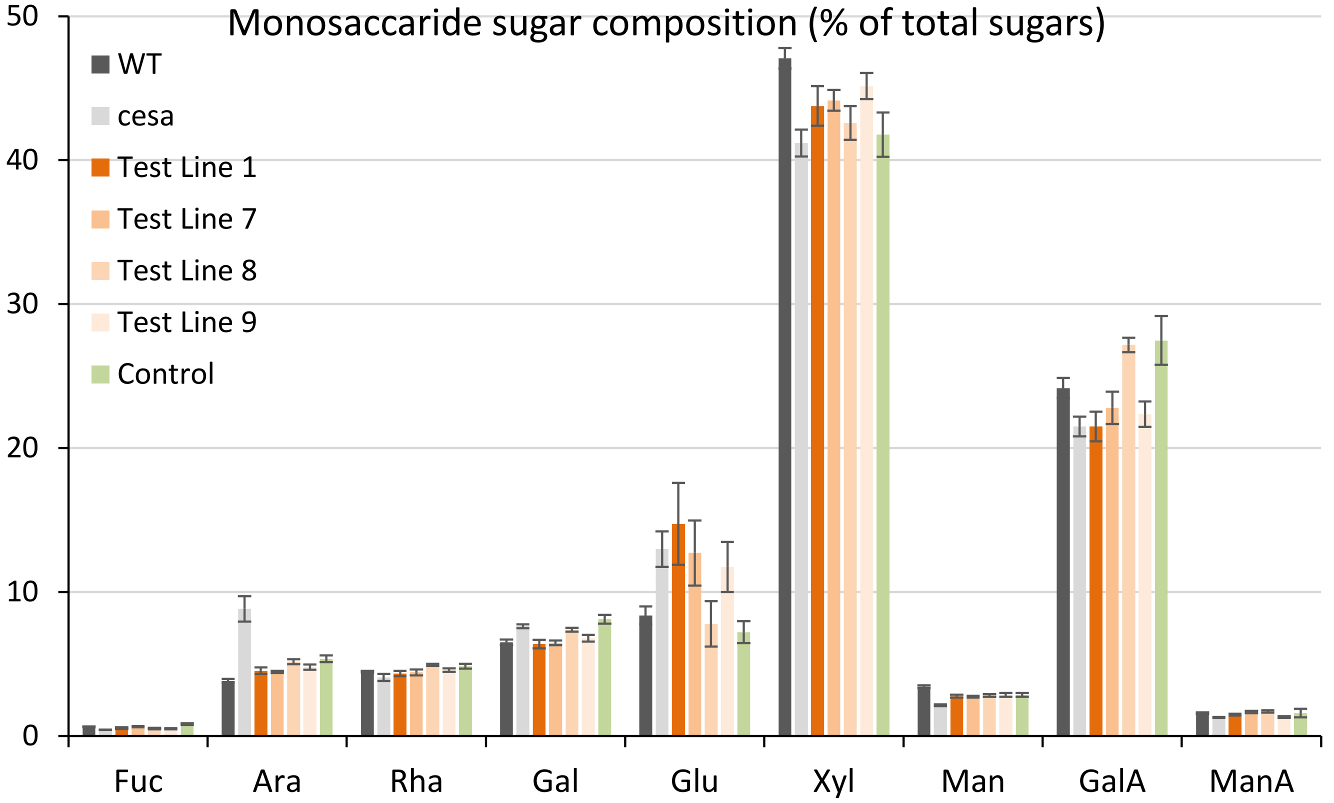

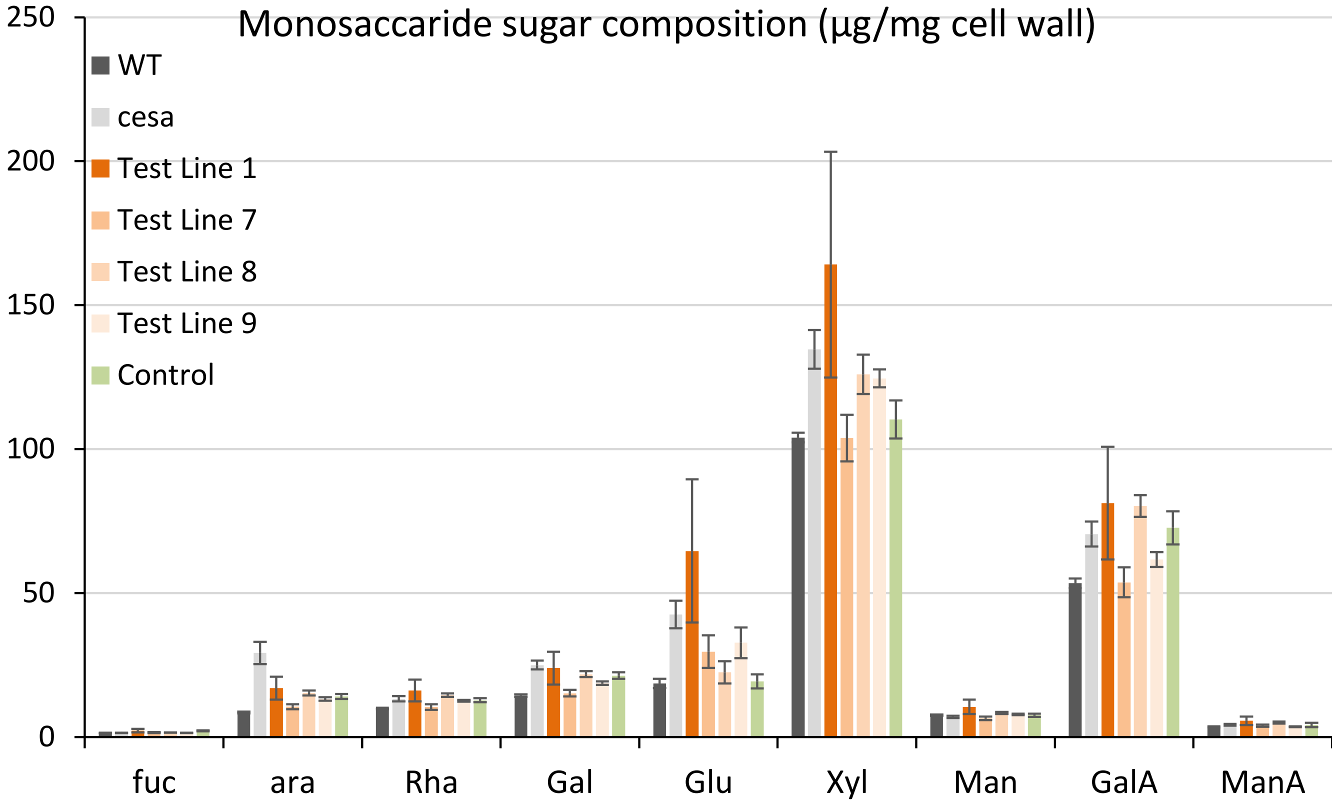

Supplementary Figure S7 Monosaccharide analysis of Arabidopsis *cesa4,7,8* triple mutant complemented with Physcomitrium genes PpCESA6 and PpCESA8.

Total sugars were released by hydrolysis with 2M TFA, were measured using HPAEC analysis on a Dionex (Thermo) ICS-6000 PAD system and expressed as percentage of total sugars. Test lines refer to 4 independent lines of cesa4,7,8 expressing PpCESA6+8. These are the same lines as in panel C. “Control” refers to a group of 4 different genotypes – PpCESA6 transformed into Arabidopsis *cesa7* mutant; PpCESA6 transformed into Arabidopsis *cesa4,7* mutant; PpCESA6 transformed into Arabidopsis *cesa7,8* mutant, and PpCESA8 transformed into Arabidopsis *cesa4* mutant. These lines were chosen because they complemented both the plant height and cellulose defect of the mutants equally well (Figures 3B, 4B, S5B, S6B).

Primer ID	Target	Primer Sequence	Used for
RM233f	PpCESA03	caaaaaagcaggctggATGGAGGCTAATGCGGGCCT	making entry clones for Physcomitrell CESAs
RM234f	PpCESA04	caaaaaagcaggctggATGAAGGCGAATGCGGGGCT	
RM235f	PpCESA05	caaaaaagcaggctggATGGAGGCTAATGCAGGCCT	
RM236f	PpCesA06	caaaaaagcaggctggATGGAGGCCAATGCGGGGTT	
RM237f	PpCESA07	caaaaaagcaggctggATGGAGGCGAATGCAGGGCT	
RM238f	PpCESA08	caaaaaagcaggctggATGGAGGCTAATGCGGGCCT	
RM233r	PpCESA03	caagaaagctgggtaTCACAAGCAGGTGAGGCCGC	
RM234r	PpCESA04	caagaaagctgggtaCTATCGACAGTTGATCCCAC	
RM235r	PpCESA05	caagaaagctgggtaCTAACAGCTAAGCCCGCACT	
RM236r	PpCesA06	caagaaagctgggtaTCAACAGTTTATCCCGCACT	
RM237r	PpCESA07	caagaaagctgggtaTCAACAGTTTATCCCGCACT	
RM238r	PpCESA08	caagaaagctgggtaTTACAAGCAGGTGAGGCCGC	
RM233af	PpCESA3rep1	accggacgcaatgctccacgggcgcatgagttatgg	repairing Physcomitrell CESAs
RM233ar	PpCESA3rep1	ccataactcatgcgcccgtggagcattgcgtccggt	
RM233bf	PpCESA3rep2	gtggggtgctgcacccaagcgatatggacctaaatgacccagaccttccaataatggatgaatcaagacaac	
RM233br	PpCESA3rep2	attggaaggtctgggtcatttaggtccatatcgcttgggtgcagcaccccaccttcgctcatcatcatttgc	
RM235af	PpCESA5rep1	caggatggcacgccatggccagggaacaacagcaga	
RM235ar	PpCESA5rep1	tctgctgttgttccctggccatggcgtgccatcctg	
RM235bf	PpCESA5rep2	agactggcgtacatcaacaccacaatctatccgttg	
RM235br	PpCESA5rep2	caacggatagattgtggtgttgatgtacgccagtct	
RM236af	PpCESA6rep1	aaccgcgagacgtatctggatcggctgtcgttgagg	
RM236ar	PpCESA6rep1	cctcaacgacagccgatccagatacgtctcgcggtt	
RM236bf	PpCESA6rep2	ccaacgttcgtgaaggagcggagagccatgaagagg	
RM236br	PpCESA6rep2	cctcttcatggctctccgctccttcacgaacgttgg	
RM237af	PpCESA7rep1	aggccactggatccggcgaaggatctgggatcgtat	
RM237ar	PpCESA7rep1	atacgatcccagatccttcgccggatccagtggcct	
RM237bf	PpCESA7rep2	aagaagttcaacatcgagccccgggcacctgaggtg	
RM237br	PpCESA7rep2	cacctcaggtgcccggggctcgatgttgaacttctt	
RM151f	SmCESA1_Sm141535	ggggacaagtttgtacaaaaaagcaggctggatggaggcgagcgcgggatt	making entry clones for Selaginella CESAs
RM151r	SmCESA1_Sm141535	ggggaccactttgtacaagaaagctgggtactagcagttcaatccacact	
RM152f	SmCESA2_Sm73698	ggggacaagtttgtacaaaaaagcaggctggatggaggcgaatgcgggcct	
RM152r	SmCESA2_Sm73698	ggggaccactttgtacaagaaagctgggtactagcagttgatgccacact	
RM153f	SmCESA3_Sm75715	ggggacaagtttgtacaaaaaagcaggctggatggaggccaacgctgggct	
RM153r	SmCESA3_Sm75715	ggggaccactttgtacaagaaagctgggtactagcagttgaggccgcatt	
RM154f	SmCESA4_Sm163575	ggggacaagtttgtacaaaaaagcaggctggatggaggcaaatgcaggtct	
RM154r	SmCESA4_Sm163575	ggggaccactttgtacaagaaagctgggtactagcagttgataccgcatt	

Supplementary Table S1. Primers used in this study

**Name**	**Accession Number**	**Sequence Source**	**Amplification source**	**Species**
ACESA_MICDE	ADE44904.1	Genbank	Gene Synthesis	*Micrasterias denticulata*
ACESA_SPISP	HAOX-2004235	1KP transcriptome	Gene Synthesis	*Spirogyra sp*
ACESA_CYLCU	JOJQ-2007927	1KP transcriptome	Gene Synthesis	*Cylindrocystis cushleckae*
ACESA_MESEN	JOJQ-2007927	1KP transcriptome	Gene Synthesis	*Mesotaenium endlicherianum*
SmCESA2	EFJ38258.1	Genbank	Seleginella genomic DNA	*Selaginella moellendorffii*
SmCESA4	EFJ38300.1	Genbank	Seleginella cDNA	*Selaginella moellendorffii*
SmCESA1	EFJ35421.1	Genbank	Gene Synthesis	*Selaginella moellendorffii*
SmCESA3	EFJ37830.1	Genbank	Gene Synthesis	*Selaginella moellendorffii*
Fern CESA	UOMY-2008646	1KP transcriptome	Gene Synthesis	*Osmunda sp*
PpCESA03	Pp3c8_7420V3.1	Phytozome	RIKEN full-length cDNA pdp10281	*Physcomitrella patens*
PpCESA04	Pp3c9_2550V3.1	Phytozome	RIKEN full-length cDNA pdp21409	*Physcomitrella patens*
PpCESA05	Pp3c2_13330V3.1	Phytozome	RIKEN full-length cDNA pdp24095	*Physcomitrella patens*
PpCesA06	DQ902547.1	Genbank	RIKEN full-length cDNA pdp16421	*Physcomitrella patens*
PpCESA07	Pp3c15_7150V3.1	Phytozome	RIKEN full-length cDNA pdp34523	*Physcomitrella patens*
PpCESA08	Pp3c3_34520V3.1	Phytozome	RIKEN full-length cDNA pdp39044	*Physcomitrella patens*

Supplementary Table S2. Accession numbers and sequence sources for 15 CESA proteins used in this study.

Genbank sequence for *Micrasterias denticulata* lacks first two amino acids (ME). These were added during gene synthesis, based on similarity to other algal CESAs.
